# Perspectives on Swedish Regulations for Online Record Access Among Adolescents With Serious Health Issues and Their Parents: Mixed Methods Study

**DOI:** 10.2196/63270

**Published:** 2025-01-27

**Authors:** Josefin Hagström, Charlotte Blease, Arja Harila, Päivi Lähteenmäki, Isabella Scandurra, Maria Hägglund

**Affiliations:** 1 Participatory eHealth and Health Data Research Group Department of Women’s and Children’s Health Uppsala University Uppsala Sweden; 2 Digital Psychiatry Department of Psychiatry Beth Israel Deaconess Medical Center Boston United States; 3 Pediatric Oncology Department of Women's and Children's Health Uppsala University Uppsala Sweden; 4 Department of Pediatrics and Adolescent Medicine Turku University Turku University Hospital Turku Finland; 5 Pediatric Oncology and Pediatric Surgery Department of Women's and Children's Health Karolinska Institute Stockholm Sweden; 6 Informatics School of Business Örebro University Örebro Sweden; 7 MedTech Science & Innovation Centre Uppsala University Hospital Uppsala Sweden

**Keywords:** health care professionals, adolescent health, patient-accessible electronic health record, electronic health record, patient portal, survey, eHealth, interviews

## Abstract

**Background:**

With the increasing implementation of patient online record access (ORA), various approaches to access to minors’ electronic health records have been adopted globally. In Sweden, the current regulatory framework restricts ORA for minors and their guardians when the minor is aged between 13 and 15 years. Families of adolescents with complex health care needs often desire health information to manage their child’s care and involve them in their care. However, the perspectives of adolescents with serious health issues and their parents have not been studied.

**Objective:**

This study aims to qualitatively and quantitatively investigate the perceived benefits and risks of ORA and the awareness of and views on ORA regulations among adolescents with serious health issues and their parents in Sweden.

**Methods:**

We used a convergent mixed methods (qualitative and quantitative) design, consisting of a survey and semistructured individual interviews with adolescents with serious health issues (aged 13-18 y) and their parents. Participants were recruited via social media and in clinics. Quantitative data were presented descriptively. Interviews were audio recorded, transcribed, and analyzed using inductive thematic content analysis.

**Results:**

The survey population included 88 individuals (adolescents: n=31, 35%; parents: n=57, 65%). Interviews were completed by 8 (26%) of the 31 adolescents and 17 (30%) of the 57 parents. The mean age of the surveyed adolescents was 16 (SD 1.458) years, and most of the parents (29/57, 51%) were aged 45 to 54 years. The surveys indicated that most of the parents (51/56, 91%) were critical of the access gap, and most of the adolescents (20/31, 65%) were unaware of the age at which they could gain access. In the interviews, adolescents and parents identified benefits related to ORA that were categorized into 6 themes (*empowering adolescents*, *improved emotional state*, *enhanced documentation accuracy*, *improved partnership and communication*, *supported parental care management*, and *better prepared for appointments*) and risks related to ORA that were categorized into 4 themes (*emotional distress and confusion*, *threatened confidentiality*, *increased burden*, and *low usability*). Adolescents’ and parents’ views on ORA regulations were categorized into 3 themes (*challenges of the access gap, balancing respect for autonomy and support,* and *suggested regulatory change*).

**Conclusions:**

In Sweden, ORA regulations and a lack of available information cause significant inconvenience for adolescents with serious health issues and their parents. Views on access age limits differed, with adolescents expressing their perceived need for independent access, while parents exhibited concerns about adolescents having ORA. The findings indicated the importance of increased education, dialogue, and flexibility to uphold confidential and consistent delivery of adolescent health care. Further exploration is needed to understand the experiences of adolescents and parents in diverse clinical and geographic contexts, as well as the perspectives of pediatric health care professionals on restrictive ORA regulations.

## Introduction

### Background

Worldwide, online record access (ORA) enables more patients and caregivers to read their health records via patient portals. Electronic health records (EHRs) often include clinical notes, laboratory test results, and medications. In the United States, the practice of sharing clinical notes with patients is referred to as “open notes” [1]. In the European Union, individuals have a right to access their health information in registries such as EHRs, under the General Data Protection Regulation. A proposed European Health Data Space will provide patients with access to their EHRs throughout Europe. A growing body of research [2] indicates that ORA benefits for adolescents and parents are similar to those for adults (eg, better recall, increased treatment adherence, and an increased sense of control [1,3,4]). Unique benefits for adolescents and parents may also include increased autonomy [5] and a supported transition from adolescent to adult health care [6,7].

Despite potential benefits, access to pediatric records is often restricted due to concerns about confidentiality, particularly during adolescence. While most young children benefit from parental proxy access, adolescents may be deterred from seeking help for sensitive health care issues if parental monitoring remains possible, leading to potential ethical concerns and a need to protect the developing autonomy of the young person [8]. As a result, 1 policy response has been to limit adolescents’ and parents’ access when the child becomes an adolescent. However, the implementation of patient-accessible EHRs (PAEHRs) for parents and adolescents differs globally [9-11]. A variety of access control practices attempting to balance parents’ and adolescents’ needs have been adopted, with approaches either based on set access limits or case-by-case assessment.

Sweden has an advanced ORA system, facilitated through the national PAEHR 1177 Journal*.* A regulatory framework implemented in 2017 grants parents default access to their child’s PAEHR from birth until the child turns 13 years of age, after which the adolescent gains their own access at the age of 16 years. Parents’ loss of access to their child’s PAEHR when the child turns 13 years of age was due to concerns that teens may refrain from seeking care for sensitive issues, such as birth control, sexual health, or mental health, if they know that their parents have access to their records. In the first regulatory framework, adolescents themselves gained access to their records only at the age of 18 years, but this age limit was later lowered to 16 years, based on the argument that most teenagers are mature enough to make informed decisions about their health at this age. More than half (50.5%) of adolescents access their records from the age of 16 years [12]. The “access gap,” when the child is aged between 13 and 15 years, a period during which neither the child nor the parent has access, has been criticized for hindering active participation and engagement in health care, especially by parents of children with serious illness [13,14]. There is an option for both guardians and minors to apply for extended access under special circumstances (such as chronic illness) when the child is aged between 13 and 15 years. The application process involves filling out and submitting a specific paper form to the health care provider, followed by a maturity assessment and approval by the health care provider’s operations manager. To approve the application, health care professionals (HCPs) assess needs and risks, as well as the minor’s level of maturity and wishes. The process must be repeated for each clinic where the minor is receiving care. However, few applications for extended access from either adolescents or parents have been observed [12]. Thus, although the restrictive policies are intended to ensure safety, their consequences for adolescents with serious health issues and their parent caregivers may be dire and have not been studied.

Several topic experts have noted the unique need for ORA among families where adolescents are undergoing treatment or have extensive contact with health care systems [15,16]. In previous research in Sweden, we found that adolescents with lower self-reported health may have less interest in being able to control who can access their records and to conceal information from relatives [17]. Indeed, many adolescents depend on their parents for health care management [6]. In addition, in an interview study, adolescents aged 13 to 17 years with cancer and blood disorders report that, after reading their records, they are better able to prepare for clinical consultations and are able to check accuracy; moreover, examining their test results makes it easier to talk to HCPs [7]. Parents of children who are critically ill report similar benefits: the ability to check accuracy [18], better understanding [18], improved recall of information [19,20], reduced anxiety [21], and an increased sense of control [21]. They also reported that ORA makes them better able to advocate for their child [18].

Parents offer complex home-based health care and provide emotional support in advocating for their child. Rather than communicating directly with HCPs, adolescents often prefer asking questions via their parents [6,7]. Furthermore, a framework developed by Ford et al [22] described how partnerships between adolescents, parents, and HCPs can improve the adolescent’s health, stating that, for example, adolescents are more likely to seek support from their parents if they are well informed.

### Study Aim

Views on policies regarding adolescents’ and parents’ ORA access are underresearched. Increasing our knowledge about adolescents who are ill and parents in caregiving roles is vital to enable the design of informed policies and education for HCPs and patient portal users, with the long-term purpose of improving adolescent health. This study aimed to investigate, both qualitatively and quantitatively, adolescents’ and parents’ views on the perceived benefits and risks of using the PAEHR, as well as their perspectives on the national ORA regulatory framework in Sweden. Our research questions (RQs) were as follows:

- RQ1: What benefits and risks do adolescents and parents perceive regarding access to adolescents’ health records for both adolescents and parents?
- RQ2: What are adolescents’ and parents’ views on and awareness of the ORA regulations governing access to adolescents’ health records?

## Methods

### Ethical Considerations

This study received ethics approval from the Regional Ethical Review Board in Uppsala, Sweden (EPN 2022/02160). Survey participants provided consent digitally, while participants recruited in the clinic provided consent in paper format. No financial incentive was offered to survey participants; however, interview participants received a gift card worth 200 SEK (approximately US $18). Data were deidentified. 

### Study Design

Data collection occurred from March 2022 to November 2023, after ethics approval was received. A convergent mixed methods (qualitative and quantitative) approach was adopted [23,24]. Mixed methods can be defined as the “concurrent collection of both quantitative and qualitative data” where data are integrated in the analysis [23]. The purpose of combining methods was to provide a breadth of data on an understudied topic, with interviews designed to facilitate a deeper understanding of the reasons underlying the quantitative results. The qualitative component is reported in accordance with the COREQ (Consolidated Criteria for Reporting Qualitative Research) [25] guidelines (Multimedia Appendix 1).

### Participants and Setting

We recruited adolescents aged 13 to 18 years with serious health issues and parents of adolescents aged ≥13 years with serious health issues with experience in accessing their child’s EHR, either having lost access or gained extended access. In this context, serious health issues refer to physical or mental health conditions that significantly affect an adolescent’s well-being and require ongoing care or intervention. Study participants were recruited via social media advertisements by patient organizations and through collaboration with Uppsala University Hospital and other clinical partners (eg, during appointments, by sending surveys via mail to former patients, and by posting study information in waiting rooms). Although citizens gain access to the 1177 Journal at the age of 16 years, adolescents aged ≥13 years were eligible to participate because they can apply for early access, and the study aimed to explore views on ORA regulations. Both parents of a child were able to participate.

The national regulatory framework for patients’ ORA was designed by Inera AB, the company managing the 1177 patient portal that houses the PAEHR 1177 Journal, and approved by the Swedish Association of Local Authorities and Regions. The record typically includes clinical notes, test results, and diagnoses, but information availability differs across Sweden’s 21 regions and affiliated HCPs (those who have agreed to provide access). No data are concealed from parental view unless an HCP actively chooses to block information access, which can occur in cases where, for example, child abuse is suspected.

### Data Collection

#### Survey

Two survey instruments were designed: one for adolescents and one for parents (refer to Multimedia Appendix 2 for the full surveys and Swedish translations). For this study, 9 (69%) of 13 questions were included from the adolescent survey and 13 (93%) of 14 questions from the parent survey, based on the study aim (Textbox 1). Parents’ awareness of ORA was not examined. The value of including patients in research has been noted previously [26,27]. Therefore, adolescents and parents were consulted for input, which was used to revise the surveys. Questions were not mandatory, except for those on inclusion criteria and contact information (in case the participant marked interest in participating in an interview). In Sweden, study participants aged <15 years must provide written parental consent, as mandated by the Swedish Act (2003:460) concerning the ethical review of research involving humans [28]. Therefore, age was a mandatory question for adolescents, along with the provision of written parental consent for those aged 13 or 14 years. In the web-based survey, adolescents could provide parental consent digitally. For participants aged ≥15 years, consent was provided by submitting the survey. The web-based survey was conducted using REDCap (Research Electronic Data Capture; Vanderbilt University) software.

**Survey questions for adolescents and parents.**

**ADOLESCENTS**

***Inclusion***

1. How old are you?

a. How do you want to provide guardian consent? (if younger than 15 years old)

***Experience with health care***

2. What types of care have you received?

***Views on and awareness of online record access (ORA)***

3. At what age do you think you will have (or when you received) online access to your electronic health record (EHR)?

4. Do you want to be able to read your patient-accessible EHR?

5. To what extent do you agree with the following statements? (views on ORA age limits)

6. If you have thoughts or comments to add about the EHR, please write below.

***Demographics***

7. You identify as...? (gender)

8. Who do you live with? (select all that apply)

9. Would you participate in an interview about this?

- E-mail
- Phone number
- How would you prefer to be contacted?

**PARENTS**

***Inclusion***

1. How old is the child for whom you base your answers?

2. Did you read your child’s EHR online before your child turned 13?

***Experience with health care***

3. What types of care has your child received?

***Views on ORA***

4. To what extent do you agree with the following statements? (views on ORA age limits)

5. If you have thoughts or comments to add about the EHR, please write below.

***Demographics***

6. You identify as...? (gender)

7. How old are you?

8. Which region do you live in?

9. In what type of area do you live?

10. What is your level of knowledge in Swedish?

11. What is your highest completed education?

12. Approximately how much is your household income before tax in a normal month?

13. Would you participate in an interview about this?

- E-mail
- Phone number
- How would you prefer to be contacted?

#### Interviews

The first author (JH, a PhD student in health informatics with past experience of qualitative research and training) conducted the interviews with participants who registered their interest in the survey. Verbal consent for audio recording was provided before the interview. Interviews were conducted between February 2022 and November 2023 via telephone or videoconferencing software. Interview guides were created based on prior work on ORA for adolescents, children, and parents (Multimedia Appendix 3) and included similar themes as the surveys. At the start of each interview, JH introduced herself and the reasons for conducting the research. JH had no prior relationship with any of the study participants.

### Data Analysis

Descriptive statistics were used to present quantitative survey data. Of the 25 interviews, 18 (72%) were transcribed by a professional company and 7 (28%) by JH. Interview analysis was conducted by JH and MH using NVivo (release 1.7.2; Lumivero). MH is a researcher in health informatics with experience of leading and conducting qualitative research. As views on ORA regulations were previously unexplored, we analyzed the interview data using thematic content analysis [29] with an inductive approach. First, JH read all transcripts to develop an understanding of participants’ responses. Next, the data were categorized into codes that were grouped into categories and themes. Definitions were refined further through discussions during meetings. Discussions of the findings among all authors improved credibility. Analysis of perceived benefits and risks was inspired by previous work [7,30].

## Results

### Participant Demographic Characteristics

In total, 31 (74%) of 42 adolescents and 57 (81%) of 70 parents completed the survey and were included in the study. While most of the participants identified as woman in both groups, the proportion was larger among parents (47/57, 83%) than among adolescents (15/31, 48%; Table 1). Regarding gender, of the 31 adolescents, 3 (10%) selected *other* or did not want to state their gender. Of the 31 adolescents, 1 (3%) had recently turned 19 years of age.

**Table 1:** Survey and interview participants’ demographic characteristics (n=88).

Characteristics		Adolescents (n=31)			Parents (n=57)	
		Interviewed (n=8), n (%)	Not interviewed (n=23), n (%)	Interviewed (n=17), n (%)		Not interviewed (n=40), n (%)
**Gender**						
	Man	4 (50)	9 (39)	3 (18)		6 (15)
	Woman	4 (50)	11 (48)	13 (76)		34 (85)
	Other	0 (0)	1 (4)	0 (0)		0 (0)
	Don’t know or don’t want to state	0 (0)	2 (9)	0 (0)		0 (0)
	Missing	0 (0)	0 (0)	1 (6)		0 (0)
**Child age (y)**						
	13	1 (13)	1 (4)	1 (6)		17 (42)
	14	0 (0)	0 (0)	3 (18)		9 (22)
	15	2 (25)	4 (17)	5 (29)		5 (12)
	16	1 (13)	4 (17)	0 (0)		2 (5)
	17	0 (0)	10 (44)	8 (47)		5 (12)
	18	3 (38)	4 (17)	0 (0)		1 (2)
	19-25	1 (13)	0 (0)	0 (0)		1 (2)
**Child’s diagnosis^a^**						
	Juvenile arthritis	2 (25)	7 (30)	4 (24)		17 (42)
	Cancer	2 (25)	9 (39)	9 (53)		10 (25)
	Gastrointestinal diseases (eg, irritable bowel syndrome)	2 (25)	3 (13)	0 (0)		5 (12)
	Mental health issues	1 (13)	3 (13)	1 (6)		4 (10)
	Diabetes	0 (0)	2 (9)	1 (6)		3 (7)
	Other	3 (38)	1 (4)	3 (18)		8 (20)

^a^Participants could select all that applied; therefore, the total can exceed 100%.

Of the 31 adolescents and 57 parents who responded to the survey, 11 (36%) adolescents and 28 (49%) parents agreed to participate in an individual interview. Ultimately, of those agreeing to take part in an interview, 8 (73%) of the 11 adolescents and 17 (61%) of the 28 parents completed an interview, while 3 (27%) adolescents and 11 (39%) parents did not participate in an interview despite registering interest in the survey due to scheduling difficulties or a lack of response. Interviews lasted for a mean of 28 (range 13-40) minutes for adolescents and a mean of 42 (range 21-70) minutes for parents. Of the 17 parents, 5 (29%) reported having a medical profession. Notably, 2 (12%) of the 17 interviewees were parents of the same child, and none was a parent of a participating adolescent. All participants reported having moderate or higher levels of digital literacy.

As shown in Table 2, of the 8 adolescents, 2 (25%; aged 13 and 15 years) preferred parental ORA, whereas the remaining adolescents (n=6, 75%; aged 15 to 19 years) wanted their own access and either did not want or were indifferent to parental access, perceiving no need for it but expressing no privacy concerns. Almost all parents (16/17, 94%) desired longer access than current regulations allow (Table 3). The exception was a parent whose access was lost when their child was diagnosed with a serious health issue after the age of 13 years. Most of the parents (10/17, 59%) did not have access to their child’s EHR; 2 (20%) of these 10 parents reported accessing it via the child logging in on their behalf. Additional demographic characteristics can be found in Multimedia Appendix 4.

**Table 2:** Adolescent participants’ characteristics as reported in the interviews (n=8).

ID	Age (y)	Gender^a^	Interview setting	Child’s diagnosis (age at diagnosis [y])	Current ORA^b^ preference					Current ORA situation
					Adolescent	Start age (y)	Parent	End age (y)		
A27	19	Female	Telephone	Juvenile arthritis (14)	Yes, in favor	14^c^-15^c^	Okay but no need	13	Access by default	
A14	18	Female	Telephone	Inflammatory bowel disease (17)	Yes, in favor	16-17^d^	No, opposed	16^c^-18^c^	Access by default	
A26	18	Male	Video	Inflammatory bowel disease (14)	Yes, in favor	13^c^-14^c^	No, opposed	13	Access by default	
A19	18	Female	Telephone	Juvenile arthritis (1.5)	Yes, in favor	15^c^	Okay, but no need	15^c^	Access by default	
A10	16	Male	Video	Cancer (7)	Yes, in favor	13^c^	Okay, but no need	18^c^	Access by default	
A29	15	Female	Video	Asthma and allergies (0), mental health (13)	Yes, in favor	12^c^-13^c^	No, opposed	12^d^-13	No access by default	
A23	15	Male	Telephone	Neurological disease (5)	No need	16	Yes, in favor	16^c^	No access by default	
A4	13	Male	Telephone	Cancer (2)	No need	15^c^-16	Yes, in favor	15^c^	No access by default	

^a^On the basis of survey responses.

^b^ORA: online record access.

^c^Less restrictive than current regulations.

^d^More restrictive than current regulations.

**Table 3:** Parent participant characteristics as reported in the interviews (n=17).

ID	Age (y)	Gender^a^	Interview setting	Child’s diagnosis (age at diagnosis [y])	Adolescent age (y)	Current ORA^b^ preference					Current ORA situation
						Adolescent	Start age (y)	Parent	End age (y)		
P22	48	Female	Video	Cancer (13)	14	No, opposed	16	Yes, in favor	+^c^	Gained extended access	
P48	50	Male	Video	Juvenile arthritis (10)	15	No, opposed	?^d^	Yes, in favor	+^c^	Gained extended access	
P52	37	Female	Video	Skin disease (0)	15	Okay, but no need	13^c^	Yes, in favor	16^c^ or 18^c^	Gained extended access	
P8	44	Female	Video	Cancer (7)	14	Okay, but no need	15^c^	Yes, in favor	+^c^	Gained partial extended access	
P15	55	Female	Video	Cancer (17)	17	Okay, but no need	18^e^	Yes, in favor	18^c^	Access to the child’s EHR^f^ via the child’s account (with assent)	
P31	48	Female	Telephone	Bone marrow disease (5)	17	Okay, but no need	16	Yes, in favor	+^c^	Access to the child’s EHR via the child’s account (with assent)	
P1	41	Female	Video	Cancer (11)	14	Yes, in favor	14^c^-15^c^	Yes, in favor	14^c^-15^c^	Applied for but not gained extended access	
P2	49	Female	Video	Cancer (4)	13	No need	16	Yes, in favor	16^c^	Applied for but not gained extended access	
P54	52	Female	Video	Juvenile arthritis (3)	17	Okay, but no need	16	Yes, in favor	18^c^	Applied for but not gained extended access	
P47	47	Female	Video	Juvenile arthritis (10)	15	Okay, but no need	16	Yes, in favor	+^c^	Extended access expired	
P5	57	Male	Video	Cancer (2)	17	Okay, but no need	16/18^e^	Yes, in favor	18^c^	No access, unaware of extended access	
P9	49	Female	Video	Cancer (4)	17	Okay, but no need	16	Yes, in favor	18^c^	No access, unaware of extended access	
P12	52	Female	Telephone	Cancer (4)	17	Okay, but no need	16	Yes, in favor	?^d^	No access, unaware of extended access	
P26	45	Female	Telephone	Dental surgery and orthopedic issues (7)	17	Yes, in favor	16-18^e^	No need	+^c^	No access, unaware of extended access	
P38	47	Female	Video	Juvenile arthritis (10)	14	Yes, in favor	15^c^	Yes, in favor	18^c^	No access, unaware of extended access	
P49	47	Female	Telephone	Juvenile arthritis (11)	15	Yes, in favor	16	Yes, in favor	16^c^	No access, unaware of extended access	
P55	54	Male	Video	Diabetes (16)	17	Okay, but no need	16	No need	13	Aware of extended access	

^a^On the basis of survey responses.

^b^ORA: online record access.

^c^Less restrictive than current regulations.

^d^Unable to specify.

^e^More restrictive than current regulations.

^f^EHR: electronic health record.

### Quantitative Findings

Almost all adolescents (29/31, 94%) reported wanting access to their records. Low knowledge about the access age limit was observed: only a little more than a third (11/31, 36%) knew that the access age limit was 16 years of age, and almost as many (10/31, 32%) incorrectly guessed it to be 13 years of age. Of the respondents aged ≥16 years, 43% (10/23) claimed that they did not have current access to their records although this is the default.

**Figure 1:**
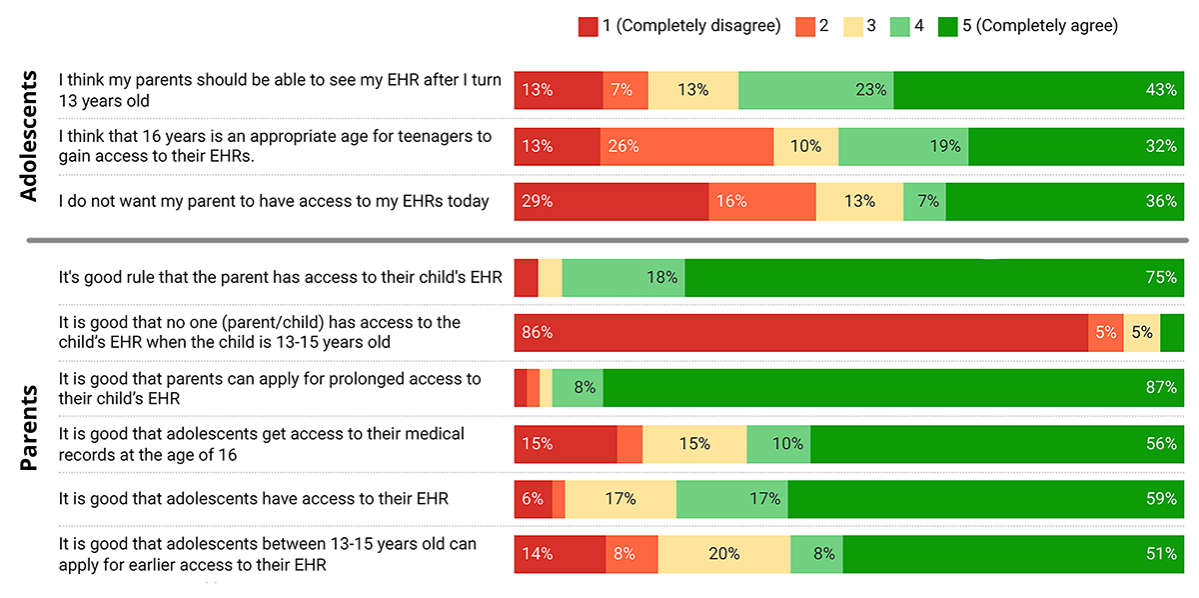
Adolescents’ and parents’ ratings for statements related to online record access and current regulations. EHR: electronic health record.

Most of the adolescents (20/31, 65%) wanted their parents to be able to read their EHR after they had turned 13 years of age (Figure 1). A slight majority (16/31, 52%) agreed that 16 years of age is an appropriate age to gain access to one’s health records. Most of the parents (51/55, 93%) were positive about parental ORA for children aged <13 years and negative about the gap in access from the ages of 13 to 15 years (51/56, 91%). Almost all parents (50/53, 94%) were positive about the option of applying for extended access for themselves, while more than half (30/51, 59%) were positive about adolescents applying for earlier access.

Most of the free-text comment data were reflected in the interviews. One parent responding to the survey noted that there should be a screening process for parents to access their children’s records, while another referred to regulations regarding parents’ rights to medication information as unclear, noting that the PAEHR is more restrictive than the information provided by pharmacies.

### Qualitative Findings

#### Perceived Benefits of ORA

Adolescents and parents reported 6 perceived benefits of ORA: *empowering adolescents, improved emotional state, enhanced documentation accuracy, improved partnership and communication, supported parental care management,* and *better prepared for appointments* (Textbox 2).

*Empowering adolescents* referred to ORA helping adolescents to gradually become more involved in their care (eg, by helping them to remember appointments, track their illness, and understand their illness history). Adolescents shared that reading the notes from the beginning helped them gain a better understanding of what they had been through, providing information about events that they were too young to remember. Both adolescents and parents envisioned that this information would be helpful when meeting new HCPs. Parents were positive about their children being able to read the records “in the future,” to understand their journey.

**Themes identified in the interviews of the perceived benefits of adolescent and parental online record access.**

**Empowering adolescents**

- “It could be good to learn how to do things when you get older. When they might not be there.” [A4, aged 13 years, diagnosed with cancer]
- “If you’re 16, I believe you have the right to receive the same information that’s actually written in the record.” [P47, mother of child aged 15 years diagnosed with juvenile arthritis]

**Improved emotional state**

- “It’s quite nice, well...if something happens, that I can go...I have security in that I can go back and read exactly everything, and even show that ‘this is how it was.’” [A19, aged 18 years, diagnosed with juvenile arthritis]
- “As a mother I have felt that it is a security to be able to go back and read, ‘what did they do now,’ and it has felt good.” [P15, mother of child aged 17 years diagnosed with cancer)

**Enhanced documentation accuracy**

- “We always have a discussion at health care meetings and such. But...they won’t write down word for word what we have said...and sometimes there are misunderstandings, and then it’s always good to be able to go back and check.” [A26, aged 18 years, diagnosed with inflammatory bowel disease]
- “At several occasions, doctors have said, ‘oh my god, how lucky you spotted that,’ and such.” [P22, mother of child aged 14 years diagnosed with cancer]

**Improved partnership and communication**

- “I have learned a lot through my parents sitting at home and reading, we have read the records together.” [A23, aged 15 years, diagnosed with neurological disease]
- “[My boyfriend] has also been allowed to read a bit from the record...So that he can gain a better understanding.” [A19, aged 18 years, diagnosed with juvenile arthritis]
- “You can sit together and reflect. Because otherwise it’s somewhat difficult to just ‘yeah, so now we’re going to talk about this’ and...then they want to go on some social media or something else like that. Maybe you can have a little focus on this.” [P54, mother of child aged 17 years diagnosed with juvenile arthritis]
- “He clams up when doctors come to talk, then he won’t speak, he won’t say anything...It may be better if we talk together in advance, and he gets to ask us his questions, and we can ask the doctor.” [P22, mother of child aged 14 years diagnosed with cancer]

**Supported parental care management**

- “You have to remember to schedule appointments and make sure to attend those appointments you’ve booked, so it’s very, very much to manage logistically when you’re sick, and it’s quite nice to get help from parents when you’re young.” [A26, aged 18 years, diagnosed with inflammatory bowel disease)
- “It has been good to track it, because then you can also tell [the child] that ‘you have to eat this vitamin because there is a deficit.’ It’s not as though I give her medication because it is fun.” [P1, mother of child aged 14 years diagnosed with cancer]
- “Just last week, we had a situation where [my child] had got a specific medication and has been taking it, but then test results came and I saw that the levels regulated by this medication were sky high, so if we had continued taking the medication it might not have been so good. Then I could contact the physician to ask ‘should we keep giving this medication or not?’ And they say, ‘no, don’t do that.’” [P22, mother of child aged 14 years diagnosed with cancer]

**Better prepared for appointments**

- “I can tell [my child] that ‘on Wednesday, we will see the physician, and we will talk about how to move forwards, and it might be that we will, blah blah blah,’ whatever it is. And then he knows, so then when we meet the physician it won’t be as dramatic.” [P22, mother of child aged 14 years diagnosed with cancer]
- “We have a younger brother and live in the countryside, we have animals, and I may have to call my work and inform them I will be gone all week, so it facilitated a lot for everyone’s well-being in the family that we could see for example test results on beforehand.” [P52, mother of child aged 15 years diagnosed with skin disease]

*Improved emotional state* referred to an increased control and a sense of safety. Adolescents and parents reported that these feelings often related to having quick access to test results and being able to go back and read information. One adolescent (A26) described reading notes from childhood as fun and nostalgic. Parents reported feeling a sense of control due to increased knowledge about the illness and its terminology. Some parents stated that access to information reduced their anxiety and worry. One parent (P52) mentioned that because physicians did not always indicate that they had seen new test results, seeing that they had been active from checking the log list provided a sense of relief. Some adolescents described feeling safe when reading the EHR with their parents or merely knowing that their parents had access. One adolescent (A23) speculated that ORA provided parents with a feeling of safety, but parents did not express this in relation to benefits of ORA for adolescents.

*Enhanced documentation accuracy* was reported as a benefit by both adolescents with ORA experience and parents; for example, it enabled them to ensure that HCPs had understood the information they shared during a consultation correctly and that there were no errors. Experiences of inaccuracies often involved HCPs misunderstanding details about symptoms, such as the degree of gravity or timing. One parent (P52) reported having once noted to an HCP that their child’s records contained someone else’s test results, apparent because the results were “too good.” Another parent (P1) who had lost access to the EHR described a case involving their child where a referral had been sent for a sex change investigation because the HCP had misunderstood the adolescent’s request to stop their period. The parent argued that if they had been able to review the EHR, they could have intervened earlier, preventing the adolescent’s distress and avoiding unnecessary efforts.

*Improved partnership and communication* was reported as a benefit by both adolescents (with or without ORA experience) and parents. This referred to ORA enabling better communication among adolescents, parents, and HCPs in working together to manage the illness. Parents stated that ORA could help adolescents formulate questions beforehand, which the parents could then forward to HCPs. Many stated that parental ORA lessened the burden on the adolescent with health issues. The youngest adolescent (A4) expressed a substantial need for parental EHR access because they felt unable to manage everything alone. Older adolescents described that their perceived need for parental EHR access had decreased over time, partly because their ability to independently communicate with HCPs had increased. In addition, ORA facilitated reading the EHR together at home, allowing families to focus on the illness and providing an opportunity to ask questions in a safe environment, either in preparation for visits or as a way to debrief afterward.

*Supported parental care management* referred to ORA facilitating parents’ provisioning of care in various ways; for example, both adolescents and parents reported that ORA facilitated parents’ management of medications and appointments, improved recall of information, and enhanced parents’ understanding of the child’s health condition. Some parents stated that the EHRs contained more information than otherwise communicated, such as positive test results. Parents described that ORA facilitated dealing with insurance tasks and that access to test results enabled them to motivate the child to take their medications. Some parents noted that reading test results allowed them to anticipate being called to the hospital, enabling them to prepare their child mentally and emotionally in advance. Quick access to test results also enabled parents to speed up the care process and prevent unnecessary distress. One parent (P52) mentioned that test results revealed the child’s actual condition, even when the adolescent claimed to feel well to avoid a hospital visit. The same parent stated that the only perceived benefit of their adolescent having ORA was that they could log in on their behalf.

*Better prepared for appointments* was reported as a benefit by both adolescents and parents, in that ORA facilitated the formulation of questions before appointments. Some parents described how ORA enabled preparations that were critical for their individual situation; for example, a parent (P52) living in the countryside could organize family life, animal care, and work in preparation for a hospital stay that might otherwise be sudden. Another parent (P22), whose adolescent had mild autism and struggled to process information during an appointment, described that ORA enabled her to use information from the EHR to prepare her child for what HCPs may bring up.

#### Perceived Risks of ORA

Participants identified 4 perceived risks of adolescent and parental ORA: *emotional distress and confusion, threatened confidentiality, increased burden,* and *low usability* (Textbox 3).

*Emotional distress and confusion* referred to the inability to understand information that was vague or written in clinical language, which could cause distress and lead to feelings of worry and frustration for both adolescents and parents. Confusion was also reported by an adolescent (A26) in relation to the unintuitive organization of information on the portal. Parents expressed concerns about adolescents reading concerning or negative test results while alone or learning about difficult medical events from their childhood, leading them to emphasize the importance (or necessity, for some) of having a parent present to answer questions and provide explanations. An adolescent aged 18 years diagnosed with juvenile arthritis (A26) recounted past experiences of feeling low after reading about traumatic experiences with health care in the EHR. Some adolescents and parents explained that their worry did not derive from accessing negative information in the record but from the progression of the illness itself. Two adolescents (A26 and A27) described that their close contact with HCPs reassured them that they would not receive bad news in the EHR without it being communicated in advance. Adolescents without experience of ORA did not anticipate emotional distress. While most desired an explanation from HCPs before receiving negative results, some reported prioritizing quick access to the information. Several parents recognized that other parents may worry; however, they themselves were not ones to worry.

**Themes identified in the interviews of the perceived risks of adolescent and parental online record access.**

**Emotional distress and confusion**

- “It’s those difficult words that one doesn’t understand...And you don’t really know what kind of test results you’re getting back or what it means, so there’s been quite a lot of Googling, consulting with mom all the time.” [A10, aged 16 years, diagnosed with cancer]
- “One day it may pop up and boom, she has cancer everywhere and they can’t do anything. And if you find out in her EHR. And the doctor hasn’t called and explained anything. Then you get a little frustrated about it.” [P2, mother of child aged 13 years diagnosed with cancer]
- “Some [parents] might say ‘no, I won’t read because it makes me more worried.’ But in our case, I felt that nothing can... I’m already worried anyway, it’s part of having a very sick child, so to speak.” [P12, mother of child aged 17 years diagnosed with cancer]
- “If I read something and suddenly just feel like...oh my god, I got heart palpitations because I read something very negative or something. Then I think, for him to sit alone without anyone beside him and read this, no, I think that would just harm him, honestly. He’s too young.” [P22, mother of child aged 14 years diagnosed with cancer]
- “Even I, who work as a medical secretary, don’t understand these terms either. I haven’t worked in oncology. I don’t understand everything either, so it’s a bit poor of the doctors then perhaps also to dictate so that one doesn’t...or the staff actually, to write and dictate so one doesn’t understand.” [P15, mother of child aged 17 years diagnosed with cancer]

**Threatened confidentiality**

- “If there’s something I don’t want them to know or something, they’ll be able to see it or they’ll be able to see all the notes that you might just...‘They don’t need to see this note.’” [A14, aged 18 years, diagnosed with inflammatory bowel disease]
- “If parents perhaps pressure one to log in, or something like that.” [A29; aged 15 years; diagnosed with asthma, allergies, and mental health issues]
- “With honor-related violence, among other things, if you see that your child perhaps has a sexual activity and you don’t believe it and so...yes, then it can get very bad...And also transgender care.” [P54, mother of child aged 17 years diagnosed with juvenile arthritis]

**Increased burden**

- “[The teen] might have to bear too much responsibility.” [P48, father of child aged 15 years diagnosed with juvenile arthritis]
- “I can understand that it could also lead to a greater workload for [HCPs], because I think that...Now, I might be a quite reasonable parent who also understands that one shouldn’t reach out unnecessarily and so on. But I can imagine that there might be others who read things and maybe don’t quite understand, and then they call.” [P22, mother of child aged 14 years diagnosed with cancer]

**Low usability**

- “The website or app itself, or how one chooses to read the records, well...I can’t say that it has a really great layout, and it’s a bit difficult to know how everything is sorted and to find things.” [A26, aged 18 years, diagnosed with inflammatory bowel disease]

*Threatened confidentiality* referred to the risk of sensitive information about the adolescent becoming visible to parents or others. While several adolescents mentioned that “the parent might see something one wants to hide,” parents commonly specified potentially sensitive topics, such as mental or sexual health. One adolescent (A26) and many parents recognized that privacy may be a problem in families where parents seek to exercise control and may not focus on the child’s best interest. Both adolescents and parents also recognized the risk that some parents might attempt to access their child’s records via their account or pressure them to log in. Some adolescents and parents mentioned the risk of young adolescents sharing information with peers or on social media.

*Increased burden* referred to a burden placed on both adolescents and HCPs. Adolescents (only those who had not had access) and parents reported that granting adolescents access could lead to excessive responsibility for adolescents, who, especially when ill, wanted to be able to depend on their parents. Some parents imagined a burden on HCPs, such as adolescents sending numerous messages through the portal. Moreover, HCPs may feel compelled to omit sensitive information from the EHR, which would be detrimental to the child’s future care. Some parents stated that patients’ ORA is not a priority for HCPs, who understandably focus on providing care.

*Low usability* was reported as a downside or risk by an adolescent (A26), who found it challenging to locate and identify specific types of notes, such as diagnoses, clinical notes, or other documentation, due to unclear categorization and sorting. As a result, they spent more time searching for the desired information.

#### Views on ORA Regulations

**Figure 2:**
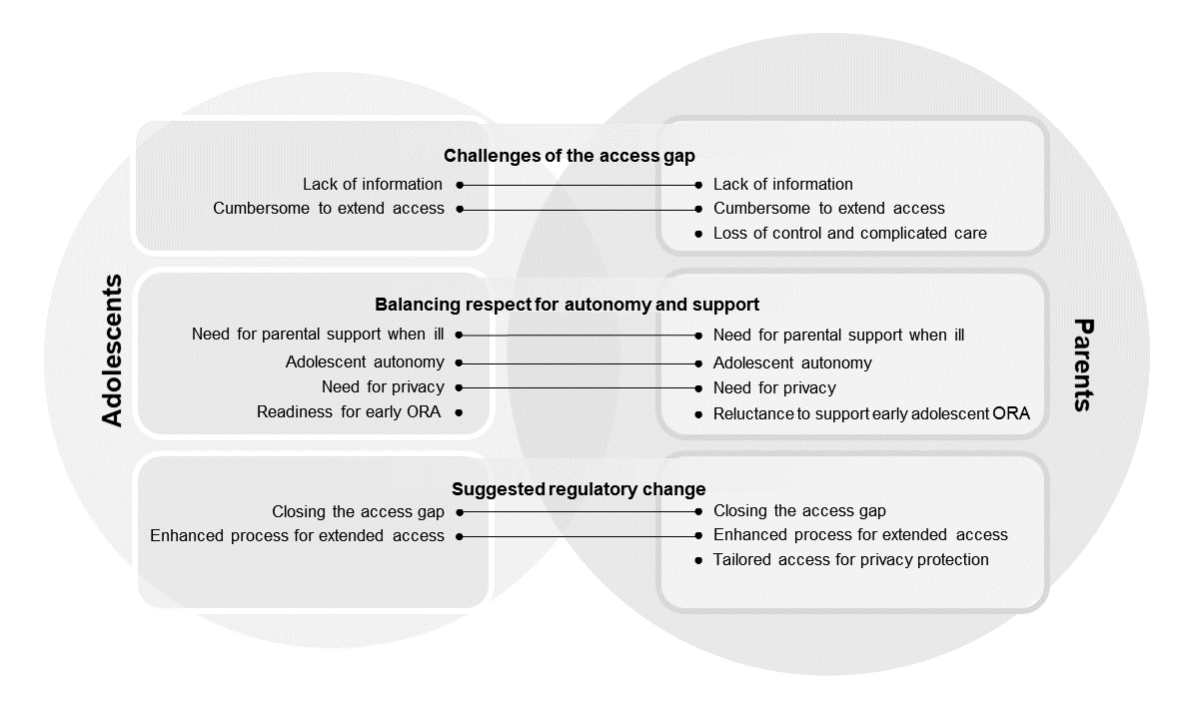
Themes related to adolescents’ and parents’ views on online record access (ORA) regulations.

Three themes were identified with regard to views on ORA regulations: *challenges of the access gap*, *balancing respect for autonomy and support*, and *suggested regulatory change* (Table 4). Similarities and differences between adolescents’ and parents’ report of themes and subthemes are visualized in Figure 2.

**Table 4:** Themes related to adolescents’ and parents’ views on online record access regulations.

Themes and subthemes		Representative quotes
**Challenges of the access gap**		
	Lack of information	“Being able to access it at all, I found out just a month ago from someone at a primary care clinic, that like, you have to order it and then you’ll get it on paper...but, that you can get earlier access or so, I haven’t heard anything about. And it’s probably because even when you ask your physician at the clinic, they don’t know either.” [A29; aged 15 years; diagnosed with asthma, allergies, and mental health issues] “We knew it would come, but we still thought that ‘she is sick, maybe they understand that we should have access to the record anyways.’ But it...it wasn’t the case.” [P2, mother of child aged 13 years diagnosed with cancer]
	Losing access causes a loss of control and complicates care	“I got really angry [when losing access], as I said, because it’s my child that I’m responsible for. Then she shouldn’t be able to do a bunch of things without my knowledge either.” [P15, mother of child aged 17 years, diagnosed with cancer] “I felt a confusion, not being able to read the test results, if she has inflammation in her body, where does she have it or does she not, how do the liver tests look? You couldn’t follow her illness in the same way when you couldn’t read the EHR [electronic health record].” [P38, mother of child aged 14 years diagnosed with juvenile arthritis] “There is extra work, both for me and physicians...or the nurse I guess, because I have to call in. And I have to match their phone hours, and then I take their time and time from those who need it better.” [P1, mother of child aged 14 years diagnosed with cancer]
	Cumbersome to extend access	“It feels like a very complicated process. Especially for 13- to 15-year-olds...like, we find it easy to do something on our mobile phones, fill it out there, but if we have to first find a paper, figure out how to print it, fill it out, send it in, get it signed, get it into the system...that probably takes a few months.” (A29; aged 15 years; diagnosed with asthma, allergies, and mental health issues] “You have a thousand other things that are higher priority when you have sick children...To then submit long paper forms, find the right nurse who also doesn’t know what to do...and specifically write the exact clinic on them when you go to...they send referrals here and there, I barely know what all the clinics he has been to and is going to are called.” [P52, mother of child aged 15 years diagnosed with a skin disease]
**Balancing respect for autonomy and support**		
	Need for parental support	“It can be good, because you’re darn young, and maybe you don’t know how to do everything yourself and might need help.” [A4, aged 13 years, diagnosed with cancer] “It’s completely different from person to person. Some might develop faster than others. And if you haven’t, it might be nice for parents to be able to help.” [A27, aged 19 years, diagnosed with juvenile arthritis] “I probably wouldn’t have wanted [adolescents’] access that early. There is a conflict, I’m thinking, with the child’s right to know and at the same time, whether a child is emotionally equipped to see serious illnesses or prognoses, and take in the information. I think that you’re young when you are...below 16, you’re still young to deal with these difficult things.” [P48, father of child aged 15 years diagnosed with juvenile arthritis] “Maybe they don’t need access before they’re 18...I feel at least that I’m glad he can access his EHR. Otherwise, we wouldn’t have seen anything. Because it’s mostly me who reads it.” [P15, mother of child aged 17 years diagnosed with cancer]
	Adolescent autonomy	“I think maybe you can have access slightly earlier, so that you can like, understand a little bit.” [A10, aged 16 years, diagnosed with cancer] “I live in [region], where many live in rural areas and when you’re 15-16, you can move away from home. Like, into the city, and then you become more or less an adult and a bit more, ‘I can take care of my record myself.’” [A29; aged 15 years; diagnosed with asthma, allergies, and mental health issues] “I would probably wish that [my daughter] had access to see her test results. It’s still her life, you know.” [P38, mother of child aged 14 years diagnosed with juvenile arthritis] “This thing about medication and making your own decisions...it’s something you have to phase in. For it to work, I think you need to phase it in over a few years. It’s not something you just fix with a bang on your 18th birthday. So it’s still good at 16 years old that they get access and...if they are mature and willing.” [P54, mother of child aged 17 years diagnosed with juvenile arthritis] “There are some children who are more mature than their parents, I think it provides an opportunity for the children who are interested and listen during their meetings like parents...I think the opportunity should be there for them to see the whole time.” [P52, mother of child aged 15 years diagnosed with a skin disease]
	Need for privacy	“It really depends, like some might start...like, if we say that one goes to BUP [child and youth mental health services] or some other place...I would say it begins at around 12-13, that one seeks help but doesn’t want to tell.” [A29; aged 15 years; diagnosed with asthma, allergies, and mental health issues] “I don’t think it’s really about age, but more about what happens in the child’s life. Because I think that there might be a difference...I’m thinking in terms of privacy. Now we have an ongoing illness process where I believe he also benefits from us being involved in and being able to help follow up. And as long as he’s in that loop...I think it’s very important that we have access. But after that, maybe not as much.” [P22, mother of child aged 14 years diagnosed with cancer] “There are far too many controlling guardians out there who want to control their children and all that. But I think that it must be more prevalent within certain areas, such as counseling support and youth clinics and so on. And I think we should never have access to those.” [P8, mother of child aged 14 years diagnosed with cancer]
**Suggested regulatory change**		
	Closing the access gap	“I think maybe parents should have access until you are 13-14, and then I think it should be brought up that you can gain your own access to it.” [A26, aged 18 years, diagnosed with inflammatory bowel disease] “I think one should be able to read always, or maybe until they’re 16, and from 16 they can perhaps log in themselves.” [P52, mother of child aged 15 years diagnosed with skin disease]
	Enhanced information on extended access	“Could one consider sending information to the health care center, to youth...I mean, to health care centers, that parents can apply [for access extension]? Or maybe conduct some webinar?” [P49, mother of child aged 15 years diagnosed with juvenile arthritis] “I think it should be very clear on 1177, that ‘if you want to see your child’s medical record, fill in here’ or something like that.” [P54, mother of child aged 17 years diagnosed with juvenile arthritis] “For anything that isn’t transient, like a cancer diagnosis that doesn’t go away on its own, there should be a dialogue at least 6 months before the child turns 13 with the treating physician, and it should pop up in the EHR when the doctor opens it. Explaining to both children and parents, ‘this is what will happen if we don’t do anything, and how do you view it and what would you like access to?’ and so on...Perhaps even that there can be some standard procedure, ‘this is how we usually do it when it comes to cancer diseases, that you have access to blah, blah, blah...this and this.’ But this you won’t see. So that the wheel doesn’t need to be reinvented every time.” [P8, mother of child aged 14 years diagnosed with cancer]
	Tailored access for privacy protection	“Can’t you block certain parts [from parents], like youth psychiatry, counseling, youth health, or something like that? I’m thinking that you could be allowed to read all the time but maybe block certain parts.” [P52, mother of child aged 15 years with skin disease] “Maybe [the adolescent] can make an agreement with the person writing in the record that ‘no, but this can be kept hidden, I don’t want anyone else to see this.’ That there’s like a toggle switch, like, private or not. You know, like when booking [setting up appointments] in Outlook, you can just be like, this is private.” [P22, mother of child aged 14 years diagnosed with cancer] “I think that if a child seeks care independently, the health care professional could perhaps ask the question ‘do you not want your parent to be able to read this?’ That it can be customized.” [P47, mother of child aged 15 years diagnosed with juvenile arthritis]

*Challenges of the access gap* were mentioned by both adolescents and parents. Several parents expressed strong feelings of frustration and desperation over losing their access, citing the many benefits they had experienced. Despite receiving notifications, several parents had not fully understood that they would lose access when the child turned 13 years of age and were convinced that HCPs would ensure continued ORA due to their situation. Losing access made it difficult for parents to track their child’s illness because they could no longer check test results, manage appointments, or monitor medications needing refills. Instead, they had to travel to the pharmacy for medications and contact HCPs to inquire about test results during designated hours, leading them to feel that they were burdening HCPs. Most adolescents and some parents were unaware of the option to apply for extended access, especially in the case of adolescents. Of those who had applied, a majority described the application process as cumbersome: identifying the right form; submitting separate applications for each desired unit; and parents needing to frequently raise the issue with HCPs, who often lacked the necessary knowledge or information. Difficulties with the application process were both anticipated and encountered. One adolescent (A26) and several parents had given up on their efforts to apply for access due to these challenges. A few parents who had applied for extended access immediately after losing access did not regain it until 6 months later, while 1 parent (P1) was still waiting for a signature, 2 years after applying.

Adolescents and parents were *balancing respect for autonomy and support* in their appraisal of appropriate access ages. Several participants perceived that the need for adolescent and parental ORA depended on factors other than age, such as the adolescents’ maturity and interest, which may not correlate with age. Adolescents and parents who advocated access earlier than the age of 16 years focused on adolescents’ rights to read their records, as well as the importance of involving interested adolescents with serious health issues in their care from an early age. By contrast, having a serious health issue was often perceived as a situation necessitating parental access beyond the age of 13 years, a view supported by 5 (62%) of the 8 adolescents and almost all parents (15/17, 88%). Parents stated that parental ORA should extend into adulthood for children with severe neurocognitive impairments. Some adolescents and most parents were positive about the idea of adolescents aged 16 years gaining access, citing that younger adolescents are often less capable of managing their care and require parental support. Still, most valued the opportunity for interested and mature adolescents to become involved in their own care before adulthood. Some parents were negative about early adolescent ORA, seeing it as a burden for the adolescent or perceiving illness-related information in the EHR as harmful. One parent (P48) stated that the age of 16 years is “too young to deal with such difficult matters,” and several stated that adolescents should have a parent present when reading the records.

Another source of divergent opinion was adolescents’ need for confidentiality. Privacy was a priority for adolescents and 1 parent (P55) who were positive about parents losing access when their child turned 13 years of age. Many referred to a difference in the timing of adolescents beginning to seek care for sensitive matters. While most parents were understanding of the need to conceal sensitive information, they still viewed their parental responsibility as critical. Most adolescents stated that they did not feel the need to conceal information from their parents, citing a relationship of openness and their parents’ prior involvement in the treatment. Nevertheless, some expressed a desire to hide nontreatment information, such as alcohol use. The exception was an adolescent aged 15 years who had experience with mental health care and was not open to parental access, stating that any information could be sensitive for an individual. Adolescents’ view of sensitive information was generally broader than that of the parents, with parents primarily focusing on mental and reproductive health. Furthermore, several parents and 1 adolescent (A26) discussed the importance of considering adolescents who may experience harm from parental ORA, such as children with controlling parents or in honor-based contexts. However, a few parents argued that such cases are rare and that the majority of adolescents benefit from parental access.

*Suggested regulatory change* was mentioned by both adolescents and parents. A common suggestion was to remove the existing access gap between the ages of 13 and 15 years, perhaps by finding a middle ground. Furthermore, participants proposed increasing education for adolescents, parents, and HCPs about the option of access extension and the application procedure. Parents suggested facilitating the procedure by digitizing the application to the patient portal and enabling a combined application for all units involved in treatment. Both adolescents and parents cited the need for HCPs to have more knowledge of the process. Some parents also stated that parental access should be tailored based on adolescents’ preferences or technological tagging of diagnoses, allowing for the concealment of sensitive information from parental view.

### Mixed Methods Comparison

Overall, the qualitative accounts of adolescents and parents largely reflected and elaborated on their ratings and views observed in the quantitative measurements. Consistent with survey findings, both adolescents and parents criticized the access gap between the ages of 13 and 15 years and expressed a desire for this gap to be closed. The qualitative findings provided insights into the perceived benefits and risks of ORA, which aided an understanding of various findings, such as parents’ preference for parental ORA over that of adolescents. Moreover, extensive challenges related to access extension were revealed.

## Discussion

### Summary of Findings

This study found that adolescents and parents were negative about the current access gap in Sweden for adolescents aged 13 to 15 years. While adolescents were largely positive about longer parental access, parents strongly advocated it. By contrast, adolescents also preferred earlier own access, which most parents opposed. The current option to extend access beyond the default was considered complicated due to a cumbersome application process and a lack of information and HCP knowledge. Perceived benefits and risks of ORA differ, revealing tensions in the respective views, particularly concerning parents’ worries about adolescents’ access.

### Comparison With Prior Work

While in alignment on many aspects related to ORA regulations, adolescents and parents differed mainly in their views on adolescents’ access. Our findings suggest a tension between adolescents and parents similar to that between patients and HCPs [31]—adolescents appreciate having access, while parents worry about adolescents’ lack of health literacy and the potential for harmful consequences. While adolescents in this study acknowledged the risk of not understanding medical terminology with ORA, they expressed minimal concern and described coping strategies to manage this challenge. However, many parents perceived a lack of interest among adolescents in reading their records, in addition to a high risk of potential harm. This is in line with previously identified parental concerns, including the possibility of adolescents misunderstanding information or reading negative test results independently [7]. These concerns are understandable, particularly during times of illness and vulnerability when parents want to ensure the best possible care for their child [32]. Nevertheless, a recent case study comparing Swedish and Finnish adolescents’ ORA use indicates that earlier access may lead to increased uptake at earlier ages [12].

The tension emerging from parents’ hesitance to support adolescent ORA may in part be a result of the perceived serious risks in the face of a lack of significant benefits. Notably, while several adolescents reported improved emotional states and 1 adolescent speculated that parents may feel safer with ORA, no parent envisioned that adolescents may experience more control or increased safety. Furthermore, parents reported many benefits that adolescents did not perceive, such as being able to prepare the child for medical appointments and explain medication needs. This is important because noncompliance with treatment in adolescents has been documented [33], where access to information is essential [32]. The difference in adolescents’ and parents’ perceived benefits indicates the difficulty in understanding another person’s perspective and experiences. Given the importance of partnerships between the adolescent patient, parent, and HCPs, noted in earlier work [22], there is potential for ORA to serve as a tool for improving such partnerships; for example, we identified that the opportunity to read EHRs together at home contributed positively to adolescents’ and parents’ experiences. A better understanding of each other’s perspectives might help mitigate concerns about harm related to a lack of health literacy and confidentiality. Both adolescents and parents recognized the benefit of adolescent access in empowering adolescents and supporting the transition into adulthood, supporting previous work [6,7].

Most of the adolescents in this study were positive about allowing parental access after the age of 13 years, recognizing that parental support lessened the burden on the child, particularly in the case of serious health issues. However, adolescents advocated a need for privacy regarding some types of information unrelated to treatment. The need for privacy regarding sensitive information was reported to increase with age and also varied depending on the type of care received. Notably, an adolescent aged 15 years with experience of mental health issues such as depression (A29) preferred restricted parental access from the age of 12 or 13 years, which was earlier than the preference expressed by adolescents with diseases such as cancer, inflammatory bowel disease, or juvenile arthritis. The adolescent also reported having moved away from home for school, which likely increased their level of independence. While most of the parents reported that they would accept not having access to sensitive information, they often referred to sensitive topics pertaining to sexual or mental health. Meanwhile, some adolescents cited allergies and experiences of bullying as potentially sensitive. Aligned with previous findings [17], adolescents stated that what is considered sensitive information can vary from person to person, indicating a need to allow adolescents to decide what should be visible to parents in the EHR. Parents suggested enabling customization of information availability, as has been recommended in earlier work [34]. An example of a system that allows customization is found in Finland, where HCPs assess each minor’s decision-making capacity and then allow those found capable to decide whether parents should have access. While such case-by-case approaches lead to a risk of increased work burden for HCPs, more research is required to explore the feasibility of customized access.

Adolescents and parents reported low knowledge about access extension, the intended solution to aid families of children with serious illness during the access gap between the ages of 13 and 15 years. It was reported that HCPs often lacked knowledge about the application process and even the possibility of extending access. Our previous research shows that <1% of adolescents aged 13-15 and their parents access the Swedish patient portal [12]. Furthermore, adolescents lacked knowledge about regulations. Adolescents have reported receiving little encouragement from HCPs to access their records [35]. Possibly, the implementation of ORA in Sweden has failed to involve and educate HCPs about the new regulations. Furthermore, given that ORA and EHR documentation are known causes for HCP job dissatisfaction and burnout [36], some HCPs may be reluctant to encourage ORA use. To improve our understanding of HCPs’ perspectives, the aim of a study conducted by the authors in parallel with this study was to examine the experiences and awareness of ORA regulations among oncology HCPs in Sweden.

### Implications

On the basis of the findings, we have summarized a number of implications that concern adolescents with serious health issues and their parents (Textbox 4).

**Implications of the findings.**

**Implications**

- Provide health care professionals (HCPs) with information on online record access (ORA) regulations related to extended access and guidance on how to facilitate the application procedure for adolescents and parents.
- Establish a plan for families with adolescents with serious illness to retain necessary ORA through a facilitated process of applying for extended access.
- Ensure clear communication to parents and adolescents about the management of sensitive information in records on the national patient portal.
- Provide comprehensive information to adolescents regarding the age limit for gaining access and the option to receive early access on the national patient portal.
- Provide comprehensive information to parents regarding ORA regulations, loss of access, and the option to extend access on the national patient portal.
- Foster dialogue between HCPs, adolescents, and parents regarding ORA and the concealment of sensitive information.
- Implement patient portal features that enable adolescents to customize the concealment of sensitive information according to their preferences.

### Limitations

This study has a number of limitations. The sample size was relatively small due to persistent recruitment difficulties, particularly in engaging adolescent participants. Future research may explore alternative recruitment strategies, such as reaching adolescents through schools or other community channels. Moreover, half of the adolescents interviewed (4/8, 50%) were aged 18 or 19 years, which likely affected their views. Among the parent participants, the majority (47/56, 84%) were women, and approximately one-third of the interview participants (5/17, 29%) had a medical background or a partner working as an HCP. Notwithstanding this limitation, unequal sample sizes are a common occurrence in mixed methods research [23]. Furthermore, our sample included adolescents with a variety of conditions; however, the focus was not on comparing experiences across groups with specific illnesses but rather on exploring broader perspectives related to ORA use. While the parents varied in education, residential area, and income, most had Swedish as their primary language (53/56, 95%). Given the role of socioeconomic factors in driving disparities in pediatric ORA adoption [37,38], future work should include minors and parents from diverse language backgrounds to capture broader perspectives. Moreover, 1 survey item included a negation (“I do not want my parents to access...”), which could have been challenging for participants to interpret and therefore to accurately respond to on a scale ranging from 1 to 5. The surveys used were designed by the authors because there are no validated questionnaires available for examining views on ORA. Finally, both adolescents who had read their records and those who had not participated in the study. As a result, some shared their expectations rather than experiences. We strove to present the findings with due consideration to this limitation because there may be differences between expectations and experiences of ORA [2]. We did not provide a demonstration for adolescents without access, which may have impeded their ability to answer anticipatory questions. However, most of the interviews (5/8, 62%) with adolescents were conducted via telephone, precluding visual demonstrations. Regarding interviews conducted via videoconferencing software, it is possible that this format influenced participants due to the potential sensitivity of the topic. In addition, technical issues such as connectivity problems or the lack of a private and secure environment could have further impacted the conversation flow. However, video interviews offer advantages over telephone interviews by allowing for the conveyance of nonverbal cues, such as facial expressions and body language, which can help foster a sense of connection and empathy between the interviewer and participant. An interview guide was followed to improve trustworthiness, and the study was reported in accordance with the COREQ guidelines.

### Conclusions

In Sweden, the regulatory framework on ORA (characterized by a default lack of access for adolescents aged 13-15 y and their parents) and a lack of available information on access extensions creates challenges for parents of adolescents with serious health issues. Both adolescents and parents desire consistent access to the EHR that considers adolescents’ growing need for privacy. While there is parental reluctance to support adolescent ORA due to concerns about potential harm and low perceived need, adolescents experience benefits from ORA that parents are not aware of, and vice versa. Informing adolescents, parents, and HCPs about experienced benefits and access regulations could improve partnerships, reduce distress, and facilitate adolescent care.

## Appendix

Multimedia Appendix 1 COREQ (Consolidated Criteria for Reporting Qualitative Research) checklist.

Multimedia Appendix 2 Surveys in English and Swedish.

Multimedia Appendix 3 Interview guides in English and Swedish.

Multimedia Appendix 4 Demographics and detailed results.

## Abbreviations

COREQ: Consolidated Criteria for Reporting Qualitative Research
EHR: electronic health record
HCP: health care professional
ORA: online record access
PAEHR: patient-accessible electronic health record
REDCap: Research Electronic Data Capture
RQ: research question
