# Supplementary material for: Perspectives on Swedish Regulations for Online Record Access Among Adolescents With Serious Health Issues and Their Parents: Mixed Methods Study
Source: JMIR Pediatr Parent. 2025 Jan 27;8:e63270. doi: 10.2196/63270 (PMC11811660; doi:10.2196/63270)
Supplement: Multimedia Appendix 1 [file pediatrics_v8i1e63270_app1.pdf]

Multimedia Appendix 1

Consolidated criteria for reporting qualitative studies (COREQ): 32-item checklist.

No	Item	Guide questions/description	Remarks	Section, subsection
**Domain 1: Research team and reflexivity**				
Personal Characteristics				
1.	Interviewer/facilitator	Which author/s conducted the interview or focus group?	Main author JH led all interviews.	Methods, data collection
2.	Credentials	What were the researcher's credentials? *E.g. PhD, MD*	JH: MSc, psychology.<br>MH: PhD.	Methods
3.	Occupation	What was their occupation at the time of the study?	JH: PhD candidate	Methods, data collection.
4.	Gender	Was the researcher male or female?	JH: female	Methods, data collection
5.	Experience and training	What experience or training did the researcher have?	JH has undergone courses in qualitative research and has previous experience with qualitative research, and analyzing data with NVivo.	Methods, data collection
Relationship with participants				
6.	Relationship established	Was a relationship established prior to study commencement?	JH had no professional-client relationship with any of the participants.	Methods, data collection
7.	Participant knowledge of the interviewer	What did the participants know about the researcher? *e.g. personal goals, reasons for doing the research*	At the start of each interview, JH introduced herself and the reasons for doing the research.	Methods, data collection
8.	Interviewer characteristics	What characteristics were reported about the interviewer/facilitator? *e.g. Bias, assumptions, reasons and interests in the research topic*	PhD student	Methods, data collection
**Domain 2: study design**				
Theoretical framework				
9.	Methodological orientation and theory	What methodological orientation was stated to underpin the study? *e.g. grounded theory, discourse analysis, ethnography, phenomenology, content analysis.*	A thematic content analysis was conducted.	Methods, data analysis
Participant selection				
10.	Sampling	How were participants selected? *e.g. purposive, convenience, consecutive, snowball*	Convenience sampling	Methods, participants and settings
11.	Method of approach	How were participants approached? *e.g. face-to-face, telephone, mail, email*	Face-to-face invitation by a professional, followed by phone/email from one of the researchers to make appointment.	Methods, participants and settings

12.	Sample size	How many participants were in the study?	25 participants were included, 17 parents and 8 adolescents	Results, general characteristics. Table 1: 'Survey respondents' demographic characteristics"
13.	Non-participation	How many people refused to participate or dropped out? Reasons?	3 (3/8) adolescents and 11 (11/39) parents did not participate despite registering interest in the survey due to scheduling difficulties or lack of response.	Results, participant demographic characteristics
Setting				
14.	Setting of data collection	Where was the data collected? *e.g. home, clinic, workplace*	Five adolescent interviews and four parent interviews were conducted via phone. The remaining were conducted via the video-conferencing software Zoom.	Results, Table 2
15.	Presence of non-participants	Was anyone else present besides the participants and researchers?	No	n/a
16.	Description of sample	What are the important characteristics of the sample? e*.g. demographic data, date*	Demographics (gender, adult/adolescent, age of adolescent, diagnosis), interview setting and acquaintance with client portal have been represented in table 2	Results (Participant demographic characteristics) table 1-2
Data collection				
17.	Interview guide	Were questions, prompts, guides provided by the authors? Was it pilot tested?	An interview guide was written by the authors and tested with HCPs. The guide was slightly revised based on their suggestions.	Methods, data collection
18.	Repeat interviews	Were repeat interviews carried out? If yes, how many?	No	n/a
19.	Audio/visual recording	Did the research use audio or visual recording to collect the data?	All interviews were audio recorded. The online meetings were video recorded as well.	Methods, data collection
20.	Field notes	Were field notes made during and/or after the interview or focus group?	No	n/a
21.	Duration	What was the duration of the interviews or focus group?	Interviews ranged between 13-38 minutes (mean 28 minutes) for adolescents and 24-55 minutes (mean 42 minutes) for parents	Methods, data collection
22.	Data saturation	Was data saturation discussed?	Yes	Methods, data analysis
23.	Transcripts returned	Were transcripts returned to participants for comment and/or correction?	No	na
**Domain 3: analysis and findings**				
Data analysis				
24.	Number of data coders	How many data coders coded the data?	Two authors (JH and MH)	Methods, data analysis
25.	Description of the coding tree	Did authors provide a description of the coding tree?	No	na

26.	Derivation of themes	Were themes identified in advance or derived from the data?	Themes were derived from the data. Benefits and risks were inspired by prior work.	Methods, data analysis
27.	Software	What software, if applicable, was used to manage the data?	NVivo v.1.7.2	Methods, data analysis
28.	Participant checking	Did participants provide feedback on the findings?	No	na
Reporting				
29.	Quotations presented	Were participant quotations presented to illustrate the themes / findings? Was each quotation identified? *e.g., participant number*	Yes, identified by parent/adolescent, age (adolescent), mother/father (parent), diagnosis of adolescent, age (adolescent), and participant ID. The participant ID number is indicated as A (adolescent) or P (parents) followed by the number of the interview.	Results
30.	Data and findings consistent	Was there consistency between the data presented and the findings?	Yes	Results
31.	Clarity of major themes	Were major themes clearly presented in the findings?	Yes	Results
32.	Clarity of minor themes	Is there a description of diverse cases or discussion of minor themes?	Yes	Results

Allison Tong, Peter Sainsbury, Jonathan Craig, Consolidated criteria for reporting qualitative research (COREQ): a 32-item checklist for interviews and focus groups, *International Journal for Quality in Health Care*, Volume 19, Issue 6, December 2007, Pages 349–357, DOI 10.1093/intqhc/mzm042
