# Supplementary material for: Perspectives on Swedish Regulations for Online Record Access Among Adolescents With Serious Health Issues and Their Parents: Mixed Methods Study
Source: JMIR Pediatr Parent. 2025 Jan 27;8:e63270. doi: 10.2196/63270 (PMC11811660; doi:10.2196/63270)
Supplement: Multimedia Appendix 2 [file pediatrics_v8i1e63270_app2.pdf]

Multimedia Appendix 2

Survey: Included questions and response alternatives

Adolescents: English translation

Theme	Question	**Response alternatives**
Background	How old are you?[ab]	13 years/14 years/15 years/16 years/17 years/18 years
Background	How do you want to provide guardian consent? (If younger than 15 years old)[ab]	My guardians sign electronically in the survey / I am filling in the survey at a clinic and have submitted a signed consent form to a HCP
ORA usage	At what age do you think you will have (or when you received) online access to your EHR?	13 years/14 years/15 years/16 years/17 years/18 years
ORA usage	Do you want to be able to read your PAEHR?	1 - Yes, 2 - No, 3 - Don't know
ORA usage	What types of care have you received?<br>(pick all that apply)	Mental health<br>Diabetes<br>Reumatic disease<br>Gastrointestinal disease (f ex IBS)<br>Cancer<br>Other
Views on ORA	To what extent do you agree with the following statements?	1 - Disagree, 2, 3, 4, 5 - Agree
	I think my parents should be able to see my EHR after I turn 13 years old and until I turn 18 years old.	
	I think that 16 years is an appropriate age for teenagers to gain access to their EHRs.	
	I do not want my parent to have access to my EHRs today.	
N/A	If you have thoughts or comments to add about the EHR, please write them below:	[text]
Background	You identify as…? [gender]	1 - Woman, 2 - Man, 3 - Other
Background	Who do you live with? Select all that applies	Parent/legal guardian<br>Sibling<br>Other relative<br>Boyfriend / girlfriend<br>I live by myself<br>Another person, specify:
Background	Would you participate in an interview about this?	1 - Yes, 2 - No

[a]Mandatory question

[b]Exclusion

Parents: English translation

Theme	Question	**Response alternatives**
Background	How old is the child for whom you base your answers?*	13 years/14 years/15 years/16 years/17 years/18 years
ORA usage	What types of care has your child received?<br>(pick all that apply)	Mental health<br>Diabetes<br>Reumatic disease<br>Gastrointestinal disease (f ex IBS)<br>Cancer<br>Other
Views on ORA	To what extent do you agree with the following statements?	1 - Completely disagree, 2, 3, 4,<br>5 - Completely agree<br>No opinion/don't want to state
	It's good rule that the parent has access to their child's EHR	
	It is good that no one (parent or child) has access to the child's EHR when the child is 13-15 years old	
	It is good that parents can apply for prolonged access to their child's EHR	
	It is good that adolescents have access to their EHR	
	It is good that adolescents between 13-15 years old can apply for earlier access to their EHR	
N/A	If you have more thoughts or comments, please write below:<br>__________	
Background	You identify as…?	1 - Woman, 2 - Man, 3 - Other
Background	How old are you?	18-24 years, 25-34 years, 35-44 years, 45-54 years, 55-64 years, 65 or older
Background	In what type of area do you live?	In a city<br>In a smaller town<br>In the countryside<br>Don't want to state
Background	What is your level of knowledge in Swedish?	First language<br>Not first language, but advanced skills<br>Not first language, but basic skills<br>No knowledge of [the language of the EHR]<br>Please describe how you use your child's EHR: ___________
Background	What is your highest completed education?	Elementary school<br>Upper secondary school<br>Vocational education and training<br>University, <= 3 years, Bachelor's degree<br>University, > 3 years, Master's degree<br>University, Doctoral degree<br>Don't have any formal education<br>Don't want to state
Background	Approximately how much is your household income before tax in a normal month?	0 - 19 999<br>20,000 - 39,999<br>40,000 - 59,999<br>60,000 - 79,999<br>80,000 - 99,999<br>More than 100,000<br>Don't want to state
Background	Would you participate in an interview about this?*	1 - Yes, 2 - No

*Mandatory question

Adolescents: Swedish original

Theme	Question	**Response alternatives**
Background	Hur gammal är du?[ab]	13 år/14 år/15 år/16 år/17 år/18 år
Background	Är du yngre än 15 år behöver du, förutom ditt eget samtycke, ha samtycke från dina föräldrar för att kunna delta i forskning.<br>Hur vill du lämna vårdnadshavares samtycke?<br>(Om yngre än 15 år)[ab]	1 - Mina vårdnadshavare signerar elektroniskt här i enkäten<br>2 - Jag fyller i enkäten på klinik och har lämnat in signerat samtycke på papper till personal
ORA usage	Vid vilken ålder tror du att man får (eller när du fick) tillgång till sin journal på internet?	13 år/14 år/15 år/16 år/17 år/18 år
ORA usage	Vill du kunna läsa din journal?	1 - Ja, 2 - Nej, 3 - Vet inte
ORA usage	Vilken/vilka sorters vård har du mottagit?<br>Du kan markera flera alternativ.	Psykisk ohälsa<br>Diabetes<br>Reumatisk sjukdom<br>Tarmsjukdom (t ex IBS)<br>Cancer<br>Annan, specificera: _____
Views on ORA	Innan du fyllt 13 år har din förälder tillgång till din journal. Mellan 13-15 års ålder har varken du eller din förälder tillgång till journalen. När du fyller 16 år får du egen tillgång till din journal.<br><br>Markera i vilken utsträckning du håller med om följande påståenden.	1 - Instämmer inte alls, 2, 3, 4, 5 - Instämmer helt<br>Ingen åsikt/vill inte uppge
	Jag tycker att mina föräldrar ska kunna se min journal efter att jag fyllt 13 år.	
	Jag tycker att 16 år är en lämplig ålder för att få tillgång till Journalen.	
	Jag vill inte att mina föräldrar ska ha tillgång till min journal idag.	
Background	Är det någon som idag har tillgång till din journal över internet (förutom vårdpersonal)?	1 - Ja, jag själv, 2 - Ja, min förälder/mina föräldrar, 3 - Ja, annan person, 4 - Nej, 5 - Vet inte
N/A	Om du har fler tankar eller kommentarer, skriv dem gärna nedan:	
Background	Du identifierar dig som…	Boy<br>Girl<br>Don't know/don't want to state Other, describe: _____
Background	Vem bor du med?<br>Välj alla som passar för dig.	Förälder/vårdnadshavare<br>Syskon<br>Annan släkting<br>Partner<br>Jag bor ensam<br>Annan
Background	Skulle du kunna tänka dig att delta i en intervju om detta? Intervjun tar ungefär 40-60 minuter och sker digitalt (via t ex Zoom). Vi kommer att spela in intervjun för att underlätta analysen, men du behöver inte synas i video. Som tack för ditt deltagande i intervju får du 200 kronor i form av ett presentkort.	1 - Ja, jag vill delta i en intervju, 2 - Nej

[a]Mandatory question
[b]Exclusion

Parents: Swedish original

Theme	Question		**Response alternatives**
Background	Hur gammalt är idag barnet för vilket du baserar dina svar?*		13 år<br>14 år<br>15 år<br>16 år<br>17 år<br>18 år
ORA usage	Läste du i ditt barns journal på nätet innan ditt barn fyllde 13 år?*		1 - Ja, 2 - Nej, 3 - Vet inte
ORA usage	Vilken/vilka sorters vård har ditt barn fått?<br>Du kan markera flera alternativ.		Psykisk ohälsa<br>Diabetes<br>Reumatisk sjukdom<br>Tarmsjukdom (t ex IBS)<br>Cancer<br>Annan, specificera:
Views on ORA	*Innan barnet är 13 år har föräldern tillgång till barnets journal. Mellan 13-15 års ålder har varken barn eller förälder tillgång till journalen. När barnet fyller 16 år får hen egen tillgång till sin journal.*<br>I vilken utsträckning instämmer du med följande påståenden?		1 - Instämmer inte alls, 2, 3, 4, 5 - Instämmer helt<br>Ingen åsikt/vill inte uppge
		Det är en bra regel att föräldern har tillgång till sitt barns journal	
		Det är bra att ingen (förälder eller barn) har tillgång till barnets journal när barnet är 13-15 år	
		Det är bra att föräldrar kan ansöka om förlängd tillgång till sitt barns journal	
		Det är bra att ungdomar får tillgång till sin journal vid 16 års ålder	
		Det är bra att ungdomar har tillgång till sin journal	
		Det är bra att ungdomar mellan 13-15 kan ansöka om tidigare tillgång till sin journal	
ORA effects	Att ha tillgång till och läsa mitt barns journal har gjort att jag...		1 - Instämmer inte alls, 2, 3, 4, 5 - Instämmer helt<br>Ingen åsikt/vill inte uppge
		Fått bättre förståelse för mitt barns sjukdom	
		Fått en känsla av kontroll	
		Kunnat följa läkarens råd kring mitt barns behandling bättre	
		Kunnat förbereda mig för läkarbesök	
		Känt mig förvirrad av vad jag läser	
		Känt större förtroende för vården	
		Oroat mig mer	
N/A	Om du har fler tankar eller kommentarer, skriv gärna nedan: __________		
Background	Du identifierar dig som...		Man<br>Kvinna<br>Vet inte/vill inte uppge<br>Annan, specificera:
Background	Hur gammal är du?		18-24 år<br>25-34 år<br>35-44 år<br>45-54 år<br>55-64 år<br>65 år eller äldre
Background	Vilken typ av område bor du i?		I en stad<br>På en mindre ort<br>På landsbygden

		Vill inte uppge
Background	Vad är din kunskapsnivå i svenska språket?	Förstaspråk<br>Inte förstaspråk, men avancerade kunskaper<br>Inte förstaspråk, men grundläggande kunskaper<br>Inga kunskaper i svenska
	Om Inga kunskaper i svenska: Beskriv hur du använder ditt barns journal.	[text]
Background	Vilken är din högsta avslutade utbildning?	Grundskola<br>Gymnasium<br>Yrkesutbildning<br>Universitet/högskola < = 3 år (kandidatexamen)<br>Universitet/högskola > 3 år (masterexamen)<br>Universitet/högskola, forskarutbildning<br>Har ingen utbildning<br>Vill inte uppge
Background	Ungefär hur stor är ditt hushålls inkomst före skatt en vanlig månad?<br>Räkna med eventuella bidrag, t ex barnbidrag, bostadsbidrag, studiebidrag mm.	0 - 19 999<br>20 000 - 39 999<br>40 000 - 59 999<br>60 000 - 79 999<br>80 000 - 99 999<br>Över 100 000<br>Vill inte uppge
Background	Skulle du kunna tänka dig att delta i en intervju om detta?<br>Intervjun tar ungefär 40-60 minuter och sker digitalt (via t ex Zoom). Vi kommer att spela in intervjun för att underlätta analysen, men du behöver inte synas i video. Som tack för ditt deltagande i intervju får du 200 kronor i form av ett presentkort.	1 - Ja, jag vill delta i en intervju, 2 - Nej

*Mandatory question
