# Supplementary material for: Perspectives on Swedish Regulations for Online Record Access Among Adolescents With Serious Health Issues and Their Parents: Mixed Methods Study
Source: JMIR Pediatr Parent. 2025 Jan 27;8:e63270. doi: 10.2196/63270 (PMC11811660; doi:10.2196/63270)
Supplement: Multimedia Appendix 3 [file pediatrics_v8i1e63270_app3.pdf]

Multimedia Appendix 3

Interview guide for adolescents

(13 years≤) who are being treated / have been treated for a serious health condition

I. Introduction

1 Hi ____ and thank you for wanting to participate in this interview. My name is [First and last name], I am a [title] at [institution] and working on this project.
2 In this interview, I would like to ask about your experiences of the records on [name of EHR service] and your parents' access to your records. We want to find out what is perceived as good and what can be improved, so that it can be managed better in the future.
3 You have already given your consent, but I just want to reiterate that everything you say will be treated confidentially and you will remain anonymous. You can cancel at any time without explaining why.
4 During the interview, you may think of something I do not ask about. As I said, there are no right or wrong answers to my questions, so just answer as you feel it is.
5 Do you have a question before we start?

II. Background questions

We will start with some background questions.

1 How old are you?
2 Who are part of your family?
   a Is it okay for me to say parent? Or what would you say?
   b Have any of your parents been more involved in your treatment?
3 Have you been diagnosed with a serious health condition? How old were you then?
4 During what period did your treatment take place / has your treatment taken place?
5 How used are you to using computers and digital services? (e.g. social media, connect with friends, watch videos, play games, discuss in forums, shop, read news, get health information)

III. Questions about the EHR

Now I thought I would ask a little about your experiences and thoughts about the records and how it works for parents and adolescents.

I also want to clarify because there are different services on [name of EHR service]. When I talk about records, I mean the function on [name of EHR service] where you can read notes that doctors and other staff have written about your care visits, but you can also read about diagnoses and test results. Do you understand what I mean?

1 How did you find out that you could see your records on the internet? When was that?
2 How long do you think your parents should be able to see your health records online?
3 What do you think about the fact that as an adolescent, you only get access to their records when you turn 16?
4 How old do you think you should be to be able to see your health records online?

IV. Extended access to EHR

During the period when the child is 13-16, it is possible for parents and adolescents to apply for extended access to the records. Now I thought I would ask a little about how you think it works.

1 It is possible for you to apply to be able to see your records online before you turn 16. Did you hear about it before this interview?
  a If so, where, when and how did you receive that information? Have you previously applied for access to your medical record?
    i. If so, why did you choose to apply for extended access? How was the process of applying for extended access? Did you discuss this with your parent? How do you feel today about having applied for an extended access?
    ii. If not, how do you feel about it? Have you considered applying?
  b If not, what do you think about it? Would it be interesting for you to apply for previous access?

2 Your parents are able apply to see your records online before you turn 16. Did you know about it before this interview?
  a If so, how do you view it? Have any of your parents applied to see your health record after you turned 13?
    i. If so, how did your parent apply? How did you find out that your parent applied for extended access? Was there anything he talked to you about?
  b If not, what do you think about it?

V. About your use of EHR

Now I will ask about your thoughts on having access to your records.

1 What's good about being able to look in your records?
  a Is there anything wrong with being able to read in the records? Example

2 How often do you use the records? (On computer, in mobile, app, website)
  a How often do you log in? When in the day?
  b Which features do you use the most? Read notes about care visits, check test results, etc.? Is there any function or information that you are missing?

3 Are you affected emotionally by being able to read your records?
  a For example, anxiety, fear, more control, more prepared for doctor visits, confusion.

4 Do you find it easy or difficult to navigate the records?
  a If difficult, is there anything in particular that is difficult to find? Does it make you look less in the records than you would otherwise have done?

5 Do you think you understand all the words used in the records? Do you understand what is written there? What do you do if you do not understand?

6 Have you ever read something in the records that you did not think was correct?
  a If so, what did you do?

7 Have you ever read anything in the records that made you very worried?
  a If yes, what did you do then?
  b What do you think about learning potentially negative information/diagnoses (f ex lab results, x-ray results) via the records?

8 Have you ever contacted support with a question about something you saw in your records?

9 Are you talking to someone about your records? (Family, friends, caregivers)
  a Have you ever shared information from your records to someone other than your parents? How and where? Why?
  b What do you think about possibilities to share information from your records on f ex social media?

c Is there any information in the records that you feel is extra sensitive and that you would not have anyone else to read?

VI. About your parents' use of your EHR

Now I will ask about your thoughts on parents' access to their child's records.

1 Do your parents have access to your medical record today?
   a If not, would you like your parents to read your records now? Why?
   b Are you worried about what your parents might read about you in your records? Why?
2 Do you think it is good if your parents can read in your records?
3 Is there any downside to having your parents read in your records?
4 How was it when your parents stopped being able to read your records?
5 Do you ever read the records with a parent?

VII. Additional comments

1 Do you have any more comments or something you are thinking of that we have not talked about?

VIII. Conclusion

1 Now we have reached the end of the interview.
2 We will now continue to talk to parents, adolescents and care staff. If or when the research is eventually published, information will be spread on these web pages (state where) so there it may appear.
   Do you want us to save your e-mail address for information when a study is published? YES/NO
   Thanks again for taking the time to participate.

Interview guide for parents

For adolescents who are / have been treated for a serious health condition

I. Introduction

1 Hi ____ and thank you for wanting to participate in this interview. My name is [First and last name], I am a [title] at [institution] and working on this project.
2 In this interview, I will ask about your experiences of having access to your child's health records on [name of EHR service]. We want to find out what is perceived as good and what can be improved, so that it can be managed better in the future.
3 You have already given your consent, but I just want to reiterate that everything you say will be treated confidentially and you will remain anonymous. You can cancel at any time without explaining why.
4 During the interview, you may think of something I do not ask about. You are free to bring it up immediately, or wait until the end of the interview at which point I will ask if you have anything to add. There are no right or wrong answers to my questions, so just answer as you feel it is.
5 Do you have a question before we start?

II. Background questions

We will start with some background questions.

1 How old are you?
2 How old is your child now whom your responses concern?
3 Has your child been diagnosed with a serious health condition?
4 When was your child diagnosed?
5 How old was your child then?
6 Do you have sole or shared custody of the child?
7 Do you have several children? If so, how many?
8 How accustomed are you to using computers and digital services? (At work, in person or related to care and treatment?)

III. Questions about the regulations for adolescents and parents' access to EHR

Now I will ask about your thoughts as a parent about the regulations for the records for adolescents.

I also want to clarify because there are different e-services on [name of EHR service]. When I talk about the records, I mean the function where you can read notes by doctors or other staff about your child's care visits, diagnoses, and test results. Do you understand what I mean? Of course, you have your own records, but today we will focus on is the records that you have access to for your child in the role of parent.

1 How did you find out that you could see your child's records on the internet? When was that?
2 How long do you think you as a parent should be able to see your child's records online?
3 What do you think about adolescents gaining access only when they turn 16?
   a How old do you think you should be to be able to see your records online?

IV. Extended access to EHR

During the period when the child is 13-15, it is possible for parents and adolescents to apply for extended access to the records. Now I will ask about your thoughts on it.

1 Do you know that it is possible for parents to apply for extended access to their child's medical records?
  a If so, where, when and how did you receive that information? Have you applied for access to your child's medical record? (or another legal guardian)
    i. If so, why did you choose to apply for previous access? What was it like to apply? Did you discuss with your child before applying? How do you feel today about having applied for an extended access?
    ii. If not, how come you (or your partner) did not apply?
  b If not, do you need continued access to the records? Will you apply? Would you have the benefit of being able to read in the child's records today if you could?
2 Do you know that it is possible for adolescents to apply for access to their medical records before they turn 16?
  a If so, has your child applied for prior access?
    i. If so, how did your child apply for extended access? Did you and your child discuss this first? How do you feel today about your child having access to their medical record earlier?
    ii. If not, how come your child has not applied?
  b If not, does your child need to be able to read in his or her records? What do you think if your child wants access to their medical record?

V. Questions about your use of the child's medical record

Now I will ask about your thoughts on parents' access to their child's records.

1 Had you looked in your child's medical record before your child became ill?
2 How did you experience losing access to your child's medical record at the age of 13?
  a Were you prepared for that to happen?
3 What do you see as the benefits and disadvantages of having access to your child's records online?
  a Feel free to describe examples.
4 How do you proceed when reading your child's records?
  a How often do you use the records? When in the day do you log in?
  b Log in to [name of EHR service]'s website or in the app?
  c Which features do you never use? E.g.: Read notes about care visits, check test results, etc.?
5 Are you affected emotionally by being able to read in your child's records?
  a For example, anxiety, fear, more control, more prepared for doctor visits, confusion.
6 Do you find it easy or difficult to navigate the records?
  a If difficult, is there anything in particular that is difficult to find? Does it make you look less in the records than you would otherwise have done?
7 Do you think you understand all the words used in the records? Do you understand what is written there? What do you do if you do not understand?
8 Have you ever read something in the records that you did not think was correct?
  a If so, what did you do?
9 Have you ever read anything in the records that made you very worried?
  a If yes, what did you do then?

b What do you think about learning potentially negative information/diagnoses (f ex lab results, x-ray results) via the records?

10 Is the records something you talk about or have talked about with other parents?

VI. Questions about your child's access to their EHR

Now I will ask about your thoughts on your child's access to the records.

1 How do you perceive your child to use the records?
   a Do you feel safe in that your child can handle the information in the records? [understand, aspects of data integrity – f ex share in social media]
2 How do you feel about your child being able to look in their records?
3 What are the benefits of your child being able to read in their records?
4 What disadvantages do you see in your child being able to read in their records?
5 Do you ever read the records with your child?

VII. Additional comments

1 Do you have any more comments or something you are thinking of that we have not talked about?
2 Do you have any comments on why you chose to participate?

VIII. Conclusion

1 Now we have reached the end of the interview.
2 We will now continue to talk to parents, adolescents and care staff. If or when the research is eventually published, information will be spread on these web pages (state where) so there it may appear.
   Do you want us to save your e-mail address for information when a study is published? YES/NO
   Thanks again for taking the time to participate.

Intervjuguide för ungdomar

(13 år≤)

I. Introduktion

1 Hej ____ och tack för att du vill delta i den här intervjun. Jag heter Josefin Hagström, är doktorand på Uppsala universitet och arbetar med det här projektet.
2 I den här intervjun skulle jag vilja fråga om dina upplevelser av Journalen på 1177.se och dina föräldrars tillgång till din journal. Vi vill ta reda på vad som upplevs som bra och vad som kan förbättras, så att det kan skötas bättre i framtiden.
3 Du har redan lämnat samtycke, men jag vill bara upprepa att allt som du säger kommer behandlas konfidentiellt och du förblir anonym. Du kan närsomhelst avbryta utan att förklara varför.
4 Efter intervjun kommer du kunna gå in på ett forum med andra unga personer som varit med i en intervju. Där kan du skriva inlägg om du kommer på något mer, eller läsa om andras tankar och känslor.
5 Under intervjun kanske du kommer att tänka på något som jag inte frågar om. Det finns som sagt inga rätt eller fel svar på mina frågor, så det är bara att svara som du upplever att det är.
6 Har du någon fråga innan vi börjar?

II. Bakgrundsfrågor

Vi ska börja med några bakgrundsfrågor.

1 Hur gammal är du?
2 Vilka ingår i din familj?
   a Går det bra att jag säger förälder? Eller vad skulle du säga?
   b Är det någon av dina föräldrar som varit mer involverad i din behandling?
3 Har du diagnosticerats med någon sjukdom? Hur gammal var du då?
4 Under vilken period pågick din behandling/har din behandling pågått?
5 Hur van är du med att använda datorer och digitala tjänster? (t.ex. sociala medier, ha kontakt med vänner, titta på videos, spela spel, diskutera i forum, inköp, läsa nyheter, få information om hälsa)

III. Frågor om Journalen

Nu tänkte jag fråga lite om dina erfarenheter och tankar om Journalen och hur den fungerar för föräldrar och ungdomar.

Jag vill också förtydliga eftersom det finns olika tjänster på 1177.se. När jag pratar om Journalen menar jag den funktion på 1177.se där du kan läsa anteckningar som läkare och annan personal skrivit om dina vårdbesök, men du kan också läsa om diagnoser och provsvar. Hänger du med på vilken jag menar?

1 Hur fick du reda på att du kunde se din journal på internet? När var det?
2 Hur länge tycker du att dina föräldrar ska kunna se din journal över nätet?
3 Vad tycker du om att man som ungdom får tillgång till sin journal först när man fyller 16 år?
4 Hur gammal tycker du att man behöver vara för att kunna se sin journal på nätet?

IV. Förlängd åtkomst till Journalen

Under perioden när barnet är 13-16 är det möjligt för föräldrar och ungdomar att ansöka om förlängd tillgång till Journalen. Nu tänkte jag fråga lite om hur du tycker det fungerar.

1 Det är möjligt för dig att ansöka om att kunna se din journal på nätet innan du fyller 16. Hade du hört talas om det innan den här intervjun?
   a Om ja, var, när och hur fick du den informationen? Har du ansökt om tidigare tillgång till din journal?
      i. Om ja, varför valde du att ansöka om förlängd tillgång? Hur var processen att ansöka om förlängd tillgång? Diskuterade du detta med din förälder? Hur känner du idag om att ha ansökt om förlängd tillgång?
      ii. Om nej, hur ser du på det? Har du funderat på att ansöka?
   b Om nej, hur tänker du om det? Skulle det vara intressant för dig att söka om tidigare tillgång?

2 Dina föräldrar kan ansöka om att kunna se din journal på nätet innan du fyller 16. Hade du hört talas om det innan den här intervjun?
   a Om ja, hur ser du på det? Har någon av dina föräldrar sökt om att kunna se din journal efter att du fyllde 13?
      i. Om ja, hur gick det till när din förälder sökte? Hur fick du reda på att din förälder sökt om förlängd tillgång? Var det något hen pratade om med dig om?
   b Om nej, hur tänker du om det?

V. Om din användning av journalen

Nu kommer jag fråga om dina tankar om att ha tillgång till din journal.

1 Vad är bra med att kunna titta i din journal?
   a Finns det något dåligt med att kunna läsa i Journalen? Exempel?

2 Hur använder du Journalen? (På dator, i mobilen, appen, hemsidan)
   a Hur ofta loggar du in? När på dagen?
   b Vilka funktioner använder du mest? Läsa anteckningar om vårdbesök, kolla provsvar, m.m.? Finns det någon funktion eller information som du saknar?

3 Påverkas du känslomässigt av att kunna läsa i din journal?
   a T ex oro, rädsla, mer kontroll, mer förberedd inför läkarbesök, förvirring.

4 Tycker du det är lätt eller svårt att hitta i Journalen?
   a Om svårt, är det något särskilt som är svårt att hitta? Gör det att du tittar mindre i Journalen än du annars hade gjort?

5 Tycker du att du förstår alla ord som används i Journalen? Förstår du vad som skrivs där? Vad gör du om du inte förstår?

6 Har du någon gång läst något i Journalen som du inte tyckte stämde?
   a Om ja, vad gjorde du då?

7 Har du någon gång läst något i Journalen som gjorde dig väldigt orolig?
   a Om ja, vad gjorde du då?
   b Hur ser du på att ta del av potentiellt negativa besked (t ex provsvar, röntgensvar) via Journalen?

8 Har du någon gång kontaktat support med en fråga om något du sett i Journalen?

9 Pratar du med någon om Journalen? (Familj, vänner, vårdpersonal)
   a Har du någon gång delat med dig av information från din journal till andra än dina föräldrar? Hur och var? Varför?
   b Vad tänker du om möjligheter att dela information från sin journal på t ex sociala media?
   c Finns det någon information i journalen som känns extra känslig och som du inte skulle vilja att någon annan tog del av?

VI. Om din förälders användning av din journal

Nu kommer jag fråga om dina tankar om dina föräldrars tillgång till din journal.

1 Har din förälder tillgång till din journal idag?
   a Om nej, skulle du vilja att din förälder kunde läsa i din journal nu? Varför?
   b Oroar du dig för vad din förälder kan läsa om dig i din journal? Varför?
2 Tycker du att det är bra om din förälder kan läsa i din journal?
3 Finns det någon nackdel med att din förälder kan läsa i din journal?
4 Hur var det när dina föräldrar slutade kunna läsa din journal?
5 Läser du någonsin i Journalen tillsammans med en förälder?

VII. Ytterligare kommentarer

1 Har du någon mer kommentar eller något du tänker på, som vi inte har pratat om?

VIII. Avslutning

2 Vi kommer nu fortsätta med att prata med föräldrar, ungdomar och vårdpersonal. Om eller när forskningen så småningom publiceras kommer information att spridas på de här webbsidorna (nämn namn) så där kan det dyka upp.
Vill du att vi sparar din e-postadress för att få information när någon studie eventuellt publiceras? JA/NEJ
Tack ännu en gång för att du tog dig tid och var med

Intervjuguide för föräldrar

till ungdomar som behandlas/har behandlats mot någon sjukdom

I. Introduktion

1 Hej ____ och tack för att du vill delta i den här intervjun. Jag heter Josefin Hagström, är doktorand på Uppsala universitet och arbetar med det här projektet.
2 I den här intervjun kommer jag fråga om dina upplevelser av att ha tillgång till ditt barns journal på 1177.se. Vi vill ta reda på vad som upplevs som bra och vad som kan förbättras, så att det kan skötas bättre i framtiden.
3 Du har redan lämnat samtycke, men jag vill bara upprepa att allt som du säger kommer behandlas konfidentiellt och du förblir anonym. Du kan närsomhelst avbryta utan att förklara varför.

5 Under intervjun kanske du kommer att tänka på något som jag inte frågar om. Du får gärna ta upp det direkt, eller vänta till slutet av intervjun då jag kommer att fråga om du har något att lägga till. Det finns inga rätt eller fel svar på mina frågor, så det är bara att svara som du upplever att det är.
6 Har du någon fråga innan vi börjar?

II. Bakgrundsfrågor

Vi ska börja med några bakgrundsfrågor.

1 Hur gammal är du?
2 Hur gammalt är ditt barn nu?
3 Har ditt barn någon sjukdom/behandlats för någon sjukdom?
4 Om ja, när blev barnet diagnosticerad?
5 Har du ensam eller delad vårdnad om barnet?
6 Har du flera barn? Om ja, hur många?
7 Hur van är du att använda datorer och digitala tjänster? (i arbete, personligen eller relaterat till vård och behandling?)

III. Frågor om regelverket för ungdomar och föräldrars tillgång till Journalen

Nu kommer jag fråga om dina tankar som förälder om regelverket för Journalen för ungdomar.

Jag vill också förtydliga eftersom att det finns olika e-tjänster på 1177.se. När jag pratar om Journalen så menar jag den funktion där du kan läsa anteckningar av läkare eller annan personal om ditt barns vårdbesök, diagnoser, och provsvar. Hänger du med på vilken jag menar? Du har såklart en egen journal, men den vi ska fokusera på idag är alltså den du haft tillgång till för ditt barn i rollen som förälder.

1 Hur fick du reda på att du kunde se ditt barns journal på internet? När var det?
2 Hur länge tycker du att man som förälder ska kunna se sitt barns journal över nätet?
3 Vad tycker du om att ungdomar får tillgång först när de fyller 16 år?
    a Hur gammal tycker du man borde vara för att kunna se sin journal på nätet?

IV. Förlängd åtkomst till Journalen

Under perioden när barnet är 13-16 är det möjligt för föräldrar och ungdomar att ansöka om förlängd tillgång till Journalen. Nu kommer jag fråga om dina tankar kring det.

1 Vet du om att det är möjligt för föräldrar att ansöka om förlängd tillgång till sitt barns journal?
  a Om ja, var, när och hur fick du den informationen? Har du ansökt om tillgång till ditt barns journal? (eller en annan vårdnadshavaren)
    i. Om ja, varför valde du att ansöka om tidigare tillgång? Hur var det att söka? Diskuterade du med ditt barn innan du ansökte? Hur känner du idag om att ha ansökt om förlängd tillgång?
    ii. Om nej, hur kom det sig att du (eller din partner) inte ansökte?
  b Om nej, har du behov av fortsatt tillgång till Journalen? Kommer du ansöka? Skulle du ha användning av att kunna läsa i barnets journal idag om du kunde?
2 Vet du om att det är möjligt för ungdomar att ansöka om tillgång till sin journal innan hen fyller 16?
  a Om ja, har ditt barn sökt om tidigare tillgång?
    i. Om ja, hur gick det till när ditt barn ansökte om förlängd tillgång? Diskuterade du och ditt barn detta först? Hur känner du idag om att ditt barn fick tillgång till sin journal tidigare?
    ii. Om nej, hur kommer det sig att ditt barn inte ansökt?
  b Om nej, har ditt barn behov av att kunna läsa i sin journal? Vad tycker du om ditt barn vill ha tillgång till sin journal?

V. Frågor om din användning av barnets journal

Nu kommer jag fråga om dina tankar om din tillgång till ditt barns journal.

1 Hade du tittat i ditt barns journal innan ditt barn blev sjukt? (om så är fallet)
2 Hur upplevde du att förlora tillgången till barnets journal vid 13 års ålder?
  a Var du beredd på att det skulle hända?
3 Vad ser du för fördelar och nackdelar med att ha tillgång till ditt barns Journal över nätet?
  a Beskriv gärna exempel.
4 Hur går du tillväga när du läser i ditt barns journal?
  a Hur ofta använder du Journalen? När på dagen loggar du in?
  b Loggar in på 1177:s hemsida eller i appen?
  c Vilka funktioner använder du? T ex. Läsa anteckningar om vårdbesök, kolla provsvar, m.m.?
5 Påverkas du känslomässigt av att kunna läsa i ditt barns journal?
  a T ex oro, rädsla, mer kontroll, mer förberedd inför läkarbesök, förvirring.
6 Tycker du det är lätt eller svårt att hitta i Journalen?
  a Om svårt, är det något särskilt som är svårt att hitta? Gör det att du tittar mindre i Journalen än du annars hade gjort?
7 Tycker du att du förstår alla ord som används i Journalen? Förstår du vad som skrivs där? Vad gör du om du inte förstår?
8 Har du någon gång läst något i Journalen som du inte tyckte stämde?
  a Om ja, vad gjorde du då?
9 Har du någon gång läst något i Journalen som gjorde dig väldigt orolig?
  a Om ja, vad gjorde du då?
  b Hur ser du på att ta del potentiellt negativa besked (t ex provsvar, röntgensvar) via Journalen?
10 Är Journalen något du pratar eller har pratat om med andra föräldrar?

VI. Frågor om ditt barns tillgång till sin journal

Nu kommer jag fråga om dina tankar om ditt barns tillgång till Journalen.

1 Hur uppfattar du att ditt barn använder Journalen?
   a Känner du dig trygg med att ditt barn kan hantera informationen i Journalen? [förstå informationen, integritetsaspekter – t ex dela i sociala media]
2 Hur känner du kring att ditt barn kan titta i sin journal?
3 Vilka fördelar finns med att ditt barn kan läsa i sin journal?
4 Vilka nackdelar ser du med att ditt barn kan läsa i sin journal?
5 Läser du någonsin i Journalen tillsammans med ditt barn?

VII. Ytterligare kommentarer

1 Har du någon mer kommentar eller något du tänker på, som vi inte har pratat om?

VIII. Avslutning

1 Nu har vi nått slutet av intervjun. Har du någon kommentar om varför du valde att vara med?
2 Vi kommer nu fortsätta med att prata med föräldrar, ungdomar och vårdpersonal. Om eller när forskningen så småningom publiceras kommer information att spridas på de här webbsidorna (nämn namn) så där kan det dyka upp.
   Vill du att vi sparar din e-postadress för att få information när någon studie eventuellt publiceras? JA/NEJ
   Tack ännu en gång för att du tog dig tid och var med
