# Supplementary material for: Perspectives on Swedish Regulations for Online Record Access Among Adolescents With Serious Health Issues and Their Parents: Mixed Methods Study
Source: JMIR Pediatr Parent. 2025 Jan 27;8:e63270. doi: 10.2196/63270 (PMC11811660; doi:10.2196/63270)
Supplement: Multimedia Appendix 4 [file pediatrics_v8i1e63270_app4.pdf]

Multimedia Appendix 4

Demographics

Adolescents

Characteristic	Interviewed (N=8)	Survey participants (N=31)
**Co-habitation status, % (n)***		
Parents	75% (6)	90% (28)
Siblings	25% (2)	19% (6)
Other relatives	0% (0)	0% (0)
Partner	0% (0)	0% (0)
I live alone	25% (2)	10% (3)
Other	0% (0)	0% (0)
**I want to be able to read my records, % (n)**		
Yes	88% (7)	94% (29)
No	0% (0)	0% (0)
I don’t know	13% (1)	6% (2)
**I have read my records, % (n)**		
Yes	75% (6)	61% (19)
No	25% (2)	32% (10)
I don’t know	0% (0)	6% (2)
**Diagnosis: Other**		Respiratory issues and allergies, neurosurgery (n=5)

Parents

Characteristic	Interviewed (N=17)	Survey participants (N=57)
**Swedish proficiency, % (n)**		
First language	76% (13)	93% (53)
Not first language, but advanced skills	18% (3)	5% (3)
Missing	6% (1)	2% (1)
**Residence area type, % (n)**		
City	47% (8)	44% (25)
Smaller town	24% (4)	32% (18)
Rural	29% (5)	21% (12)
Doesn’t want to state	0% (0)	2% (1)
Missing	0% (0)	2% (1)
**Education level, % (n)**		
High school	35% (6)	26% (15)
University <= 3 years	12% (2)	19% (11)
University >3 years	29% (5)	28% (16)
University, research	12% (2)	7% (4)
Vocational	12% (2)	19% (11)
**Household income, % (n)**		
0 - 19 999	12% (2)	4% (2)
20 000 - 39 999	12% (2)	23% (13)
40 000 - 59 999	6% (1)	11% (6)
60 000 - 79 999	18% (3)	19% (11)
80 000 - 99 999	24% (4)	23% (13)
Over 100 000	18% (3)	12% (7)
Doesn’t want to state	6% (1)	7% (4)
Missing	6% (1)	2 (1)
**Diagnosis: Other**		Asthma, allergies, migraine, blood pressure, dental care, vertigo, headache, fainting, skin issues, hand surgery, dietist, general care, acne (n=11)

Results

Adolescents' and parents' views on ORA regulations. 1=Completely disagree, 5=Completely agree.

**Statement, % (n)**[a]	**1**	**2**	**3**	**4**	**5**	Don't know	Missing
Adolescents (N=31)							
I think my parents should be able to see my EHR after I turn 13 years old and until I turn 18 years old.	13% (4)	7% (2)	13% (4)	23% (7)	43% (13)	0	1
I think that 16 years is an appropriate age for teenagers to gain access to their EHRs.	13% (4)	26% (8)	10% (3)	19% (6)	32% (10)	0	0
I do not want my parent to have access to my EHRs today.	29% (9)	16% (5)	13% (4)	7% (2)	36% (11)	0	0
Parents (N=57)							
For children under age 13, it's good rule that the parent has access to their child's EHR	4% (2)	0% (0)	4% (2)	18% (10)	75% (41)	1	1
It is good that adolescents have access to their EHR	6% (3)	2% (1)	17% (9)	17% (9)	59% (32)	2	1
It is good that no one (parent or child) has access to the child's EHR when the child is 13-15 years old	86% (48)	5% (3)	5% (3)	0% (0)	4% (2)	0	1
It is good that parents can apply for prolonged access to their child's EHR	2% (1)	2% (1)	2% (1)	8% (4)	87% (46)	3	1
It is good that adolescents between 13-15 years old can apply for earlier access to their EHR	14% (7)	8% (4)	20% (10)	8% (4)	51% (26)	5	1

Note: Percentages were calculated by excluding missing data and "don't know" responses.

Adolescents' awareness of regulations and interest in views on ORA. (N=31)

Question, % (n)	Response
At what age do you think you will have (or when you received) online access to your EHR?	
13 years	32% (10)
14 years	3% (1)
15 years	23% (7)
16 years	36% (11)
17 years	0% (0)
18 years	7% (2)
Do you want to be able to read your PAEHR?	
Yes	94% (29)
No	0% (0)
Don't know	6% (2)

Adolescents' reasons for reading health records online.

**I read my health record online…, n** (%)	**Yes**	**No**	**Missing**
… to get an overview of my medical history and/or treatment	14 (66.7)	7 (33.3)	10
… out of general curiosity	13 (61.9)	8 (38.1)	10
… to be sure I understood what the physician/healthcare professional said	12 (57.1)	9 (42.9)	10
… to remember the care plan/follow my treatment recommendations	8 (38.1)	13 (61.9)	10
… to be more involved in my care	5 (23.8)	16 (76.2)	10
… to prepare myself for a consultation or hospitalization	6 (19.4)	15 (71.4)	10
… because I suspect inaccuracies	3 (14.3)	18 (85.7)	10
… because I am not sure if I got the right care	2 (9.5)	19 (90.5)	10
